# An Open-Source Framework for Automated High-Throughput Cell Biology Experiments

**DOI:** 10.3389/fcell.2021.697584

**Published:** 2021-09-24

**Authors:** Pavel Katunin, Jianbo Zhou, Ola M. Shehata, Andrew A. Peden, Ashley Cadby, Anton Nikolaev

**Affiliations:** ^1^Fresco Labs, London, United Kingdom; ^2^Information Technologies and Programming Faculty, ITMO University, St. Petersburg, Russia; ^3^Department of Biomedical Sciences, University of Sheffield, Sheffield, United Kingdom; ^4^Department of Physics and Astronomy, University of Sheffield, Sheffield, United Kingdom

**Keywords:** imaging, automation, calcium imaging, immunolabeling, open-source hardware, high-throughput

## Abstract

Modern data analysis methods, such as optimization algorithms or deep learning have been successfully applied to a number of biotechnological and medical questions. For these methods to be efficient, a large number of high-quality and reproducible experiments needs to be conducted, requiring a high degree of automation. Here, we present an open-source hardware and low-cost framework that allows for automatic high-throughput generation of large amounts of cell biology data. Our design consists of an epifluorescent microscope with automated XY stage for moving a multiwell plate containing cells and a perfusion manifold allowing programmed application of up to eight different solutions. Our system is very flexible and can be adapted easily for individual experimental needs. To demonstrate the utility of the system, we have used it to perform high-throughput Ca^2+^ imaging and large-scale fluorescent labeling experiments.

## Introduction

Deep learning and artificial neural networks (ANNs) developed in the past decade have been proven useful for image analysis, optimization tasks, and robotics (LeCun et al., 2015; Hinton, 2018; Hinton et al., 2019). They are also becoming increasingly popular in solving biological problems. For example, ANN-based algorithms of cell segmentation are more accurate and much faster than conventional methods (Hilsenbeck et al., 2017). Deep learning also helps to detect transformed cells in human tissues (Van Valen et al., 2016; Coudray et al., 2018), optimize treatment conditions (Kusumoto and Yuasa, 2019), and explain animal behavior (Heras et al., 2019). Recently, an online platform has been developed to allow researchers without any prior knowledge of deep learning to use it in their own applications (von Chamier et al., 2020), further increasing the usefulness of deep learning as an analytical tool.

One important consideration when applying deep learning and other machine learning methods is the size of the training datasets. Typically, deep learning requires thousands to tens of thousands of data points (O’Mahony et al., 2019). This is often not feasible in biological experiments as they often take a long time to conduct. As a consequence, there is demand for automated systems that can perform hundreds or thousands of experiments with minimal human supervision. Such automation systems should allow for (a) single-cell microscopy (bright-field and/or fluorescent) in multiple wells (i.e., possess an XY stage); (b) automatic application of a number of different solutions; and (c) automated online analysis (e.g., cell segmentation and calculation of average brightness).

Commercially available fluorescent microscopes (e.g., Olympus BX61, Nikon Ti Widefield or Nikon A1 confocal systems, and live-cell imaging systems, such as Echo Revolve or Sartorius Incucyte) are often equipped with an XY stage that allows for multi-well fluorescent imaging. However, these systems are expensive (£15,000–£150,000) and offer any only limited automated solution application capabilities. This is allowed by many commercially available systems, such as Hamilton or Andrew, but these are difficult to incorporate with live imaging due to their size and cost.

Development of 3D printing as well as cheap electronic devices, such as Arduino and Raspberry Pie has led to a revolution in custom-building of affordable scientific equipment that earlier could only be available in big laboratories or university facilities. This equipment is not only cost-effective but also customizable for individual laboratory needs. One example of such technology is labware.net developed by Baden et al. (2015) and Maia Chagas et al. (2017), allowing 3D printing of extremely cheap lab equipment ranging from standard usable micropipettes and micromanipulators to fluorescent microscopes and optogenetic solutions.

Several open-source high-quality microscopes have been recently developed. For example, Diederich et al. (2020) have developed a customizable 3D printed open-source framework that allows for building a wide range of microscopes: from simple bright-field microscopes with autofocusing to more sophisticated systems with optical sectioning of the sample. However, these resources lack open-source systems for scanning a large number of samples. This was addressed by Sharkey et al. (2016) for small movements and by Merces et al. (2021) for robust imaging of multiwell plates. These solutions, however, do not offer any cell manipulation, although open-source liquid handling solutions have been recently developed (e.g., Wijnen et al., 2014; Almada et al., 2019; Amarante et al., 2019; Booeshaghi et al., 2019; Samokhin, 2020; Baas and Saggiomo, 2021).

Here, we present an open-source system that combines high-throughput microscopy in multiwell plates, automated solution application, simultaneous fluorescent imaging, and image analysis. It is low cost (£400–600 without and £2,500 with the fluorescent microscope), is fully customizable, and allows for up to 96 or 384 experiments to be performed, sequentially or, if experiments do not require a high sampling rate (e.g., 1 frame per minute or more), simultaneously. The platform is equipped with a 1-channel epifluorescent microscope head, which can be used to image dynamic fluorescent reporters (e.g., GCaMP and synthetic calcium dyes; Razlivanov et al., 2018) and/or samples labeled with fluorescent antibodies or dyes. We demonstrate how our system allows generating cell-biological data rapidly, under tightly controlled and reproducible experimental conditions, and at large scale.

## Results

A typical cell biology experimental paradigm often involves treatment of cells with bioactive compounds (e.g., growth factors, calcium mobilizing agonists, and cytotoxic agents) and monitoring cell behavior using fluorescent reporters or fixing cells for subsequent immunofluorescent labeling or gene expression profiling. To automate such experiments, we have developed an experimental platform that allows automatic imaging of a 96-well plate and application of eight solutions using syringe pumps. We first introduce the platform and showcase its applicability and then describe hardware, software, and systems performance in more detail.

### The Automation Platform and Its Applicability

**FIGURE 1:**
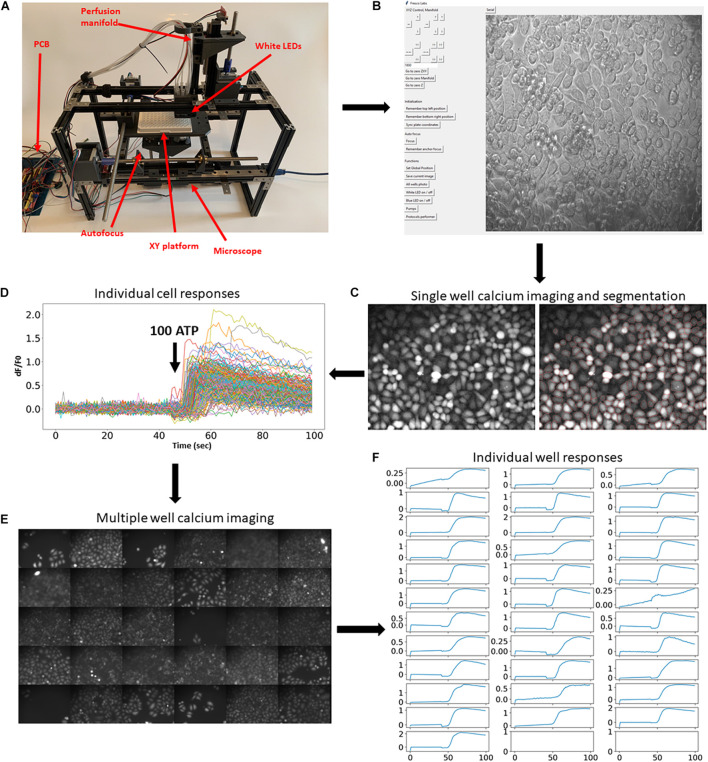
Implementation of the developed platform and its applicability in imaging fluorescent reporters. **(A)** The developed hardware and the circuit board. The key modules are assigned in red. **(B)** Hardware operating software. Top buttons operate movement of the multiwell plate, perfusion manifold, and the focusing system. Middle set of buttons drive movement of all motors to zero position. Bottom buttons operate the camera, white and blue LEDs, and pumps, and run selected protocols. **(C,D)** Example of a single calcium imaging experiment. Cells were automatically labeled with Fluo4-AM calcium dye and imaged before and after application of 100 μm ATP. Right graph shows cell segmentation using Cellpose algorithm (cell borders are highlighted in red). **(E,F)** The same calcium imaging experiment was repeated 30 times and average responses in multiple cells in each individual well were calculated and shown in panel **(E)**. Application of 100 μm ATP robustly evoked elevation of calcium concentrations in all wells. Scale bars in panels **(C,E)** are 100 μm.

The fully assembled system is shown in Figure 1A. The hardware consists of several principal modules: the X-Y stage moving a multiwell plate horizontally; a small epifluorescent microscope with autofocusing system (Supplementary Video 1); and a perfusion manifold performing application of eight solutions into individual wells (Supplementary Video 2). Building instructions and all files for 3D printing are available at https://github.com/frescolabs/FrescoM (see also Supplementary Text). The hardware can be operated using a Python written User Interface (Figure 1B, see section “Software” for more details) that controls the platform, objective, and manifold as well as autofocusing, exposure, illumination, and overall management of the experimental protocols.

To showcase this platform, we first demonstrate how it can be used in large-scale fluorescent imaging experiments (Nasu et al., 2021). A typical experimental protocol involving fluorescent reporters requires imaging cells for some time before and after application of agonists, growth factors, or other active compounds. To demonstrate the usability of the developed experimental platform for such an experimental paradigm, we performed calcium imaging using synthetic fluorescent indicators of calcium concentration. Cells were automatically labeled with fluorescent calcium dye Fluo4-AM (Figure 1C) and subjected to calcium imaging in response to 100 μm ATP. The cells were then automatically segmented (Figure 1C, right) using Cellpose algorithm (Stringer et al., 2021) and the fluorescence dynamics of individual cells were extracted (Figure 1D). The same experimental procedure and analysis was then automatically repeated in 30 other wells (Figures 1E,F and Supplementary Video 3). These experiments demonstrate that the developed platform allows for robust and automatic high-throughput imaging of fluorescent reporters.

**FIGURE 2:**
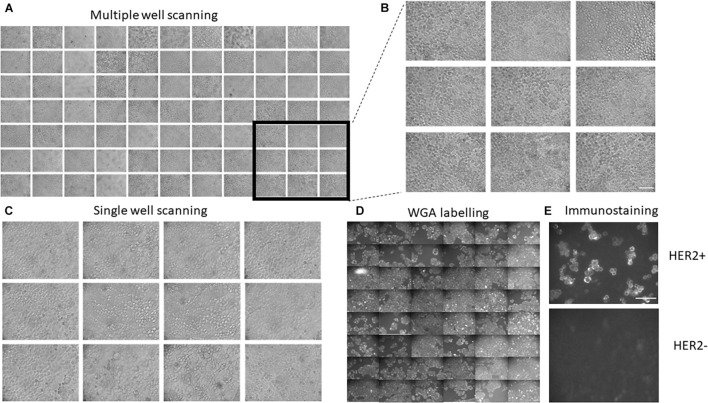
Further examples demonstrating the applicability of the developed platform. **(A,B)** Generation of a large number of images from multiple wells. **(C)** Single-well scanning. The XY platform is positioned in the center of a single well and multiple images of images of HeLa cells were taken from the same well. **(D)** Examples of automatic labeling of HeLa cells with Wheat Germ Agglutinin and subsequent automatic imaging. Panel shows examples of imaging from 48 different wells. Autofocusing was performed in white light. **(E)** Example of immunostaining experiment using Herceptin as primary antibody and Alexa 488-conjugated antihuman antibody. Strong immunolabeling was observed in SKBR3 cells but not in the HeLa cells that have relatively low levels of the receptor. Scale bars are 100 μm.

Another important advantage of the developed system is that it allows for a large-scale generation of images in a large number of wells. To demonstrate this usability, we have generated a Python protocol class (Supplementary Protocol 1, see section “Software” for more details) that makes the platform move over all 96 wells, perform focusing on each cell, and capture bright-field or fluorescent images. Example of such an experiment is shown in Figures 2A,B. Importantly, the system allows for scanning single well and make multiple images of the same well (Figure 2C), which will be useful for finding rare cells (e.g., cells undergoing mitosis/apoptosis or positive cells when transfection efficiency is low).

To demonstrate the usability of the developed experimental platform in labeling experiments, we used fluorescent Wheat Germ Agglutinin (WGA) that highlights cell membranes. Five rows of wells (48 wells altogether) were automatically washed with PBS and then loaded with a solution containing 5 mg/ml of fluorescent WGA. After 10 min at room temperature, the WGA was washed out with PBS and subjected to fluorescent microscopy. The resulting fluorescent images are shown in Figure 2D. The automatic labeling produces clear images of cells with well-defined plasma membranes, thus showing that routine labeling procedures can be automated using the developed platform.

Finally, we also tested whether the developed platform can be used for immunofluorescent staining using the anti-HER2 antibody (Herceptin) as primary antibody and Alexa 488-conjugated secondary antibody. Wells containing either SKBR3 cells (high HER2 expression) or HeLa (low HER2 expression) were automatically perfused with PBS and Herceptin. After 40 min of incubation at room temperature, cells were perfused with PBS and then secondary antibodies. After another 30 min of incubation at room temperature, the wells were perfused with imaging solution and subjected to fluorescent imaging (Figure 2E and Supplementary Figure 1). The resulting images show clear labeling of cell membranes in SKBR3 cells, which have high levels of HER2, but not HeLa cells, which have low levels of the receptor, thus demonstrating the robustness of the automatic labeling procedure.

These examples demonstrate the broad usability of the developed experimental platform in automation of the different types of cell biology experiments. Below, we describe the platform in detail and demonstrate its performance in a series of tests.

### Hardware Design

**FIGURE 3:**
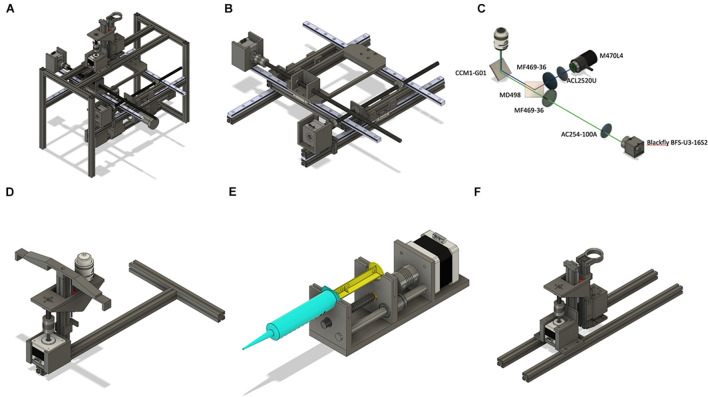
Overview of the hardware design. **(A)** Assembled hardware consists of three modules: XY platform moving in horizontal directions; perfusion manifold moving up and down, epifluorescent microscope with a Z-stage. **(B)** The XY-stage consists of two side frames made of aluminum extrusions. Two long horizontal extrusions hold MGH12 rails forming the *x*-axis. A Nema 17 motor is attached to the side frame and moves the plate along the *x*-axis. Rotation of the stepper motor moves the leading screw that is attached to either the top platform or to a plate holder. The Y stage is based on an aluminum frame holding the plate holder. The *y*-axis motor is attached directly to one of the rails. **(C,D)** Optical design of the microscope and the autofocusing system. Numbers represent Thorlabs (all optics) and FLIR (camera) item numbers. The system implements a standard inverted fluorescent microscope with the objective attached separately to a vertically oriented linear actuator driven by a Nema 17 motor **(D)**. **(E)** Design of a syringe pump. The main platform holds one Nema 17 motor, lead screw, and an 8-mm rod. The moving part represents a holder for a nut and linear bearing. **(F)** Perfusion manifold for eight syringe pumps. Manifold holds eight pipette tips with one connected to the peristaltic pump. The latter is located slightly higher than others, which helps to keep the volume in each well constant. The manifold is fixed on a linear actuator and is driven by a Nema 17 motor. The whole system is fixed on two long horizontal extrusions attached to the top of the main frame. Four white LEDs connected in series are attached to the bottom of the manifold to provide bright-field microscope functionality.

The overall structure is built with MakerbeamXL 15 mm × 15 mm extrusions connected either by L- and T-shaped aluminum brackets or by 3D printed parts. We found that using aluminum extrusion frame instead of fully 3D printed parts (both models available on https://github.com/frescolabs/FrescoM) makes the whole system more stable and reduces the vibration (data not shown). The frame consists of eight side extrusions (four vertical 300 mm and three horizontal 200 mm, two at the top and one at the bottom, Figure 3A). The sides are connected with two 400-mm extrusions holding MGH12 rails driving the *x*-axis. The *y*-axis (Figure 3B) resides on a square frame constructed of four 200-mm extrusions and connected to the *x*-axis by two 3D printed holders. Two MGH12 rails are positioned on the frame and hold one Nema 17 motor and a 96-well plate holder (Figure 3B, y_plate_holder.stl file).

The perfusion manifold (Figure 3F) consists of a holder for eight gel loading tips attached to flexible tubing *via* luer connectors. One of the tips is connected to a peristaltic pump. It is located higher than the application tips, thus providing constant solution height in each well. Alternatively, the peristaltic pump tip can be placed lower than others, thus providing more efficient washout of solution with a smaller volume. Solution change in individual wells is achieved by perfusing the wells with three to five well volumes or removal of the old solution and subsequent addition of a new solution.

The perfusion manifold is connected to a vertical 150-mm extrusion, which is connected to a vertically oriented linear actuator consisting of MGN12 rail, T8 lead screw, and a Nema 17 motor. The actuator is attached to two horizontal extrusions. Standard syringe pumps (Figure 3E) are used for solution application. The manifold is designed to have a modular structure—other modules can be attached below, above, or instead of the perfusion module. For example, we have designed a set of four white LEDs to be attached at the bottom of the manifold for bright-field microscopy as well as a holding ring for additional tubing attached at the top (Supplementary Figure 2). The LEDs are located vertically and, for high-quality image, require the manifold to be in the highest position. However, when manifold is at low position (zero distance from the LEDs and the top of the 96-well plate), cells can still be visible with higher exposure of the camera. Other modules, such as an electrode holder for simple electrophysiological experiments, holder for a miniature light guide for spatially controlled optogenetics experiments, or minipumps for individual cell manipulation, can be designed and integrated for additional experimental customization.

**FIGURE 4:**
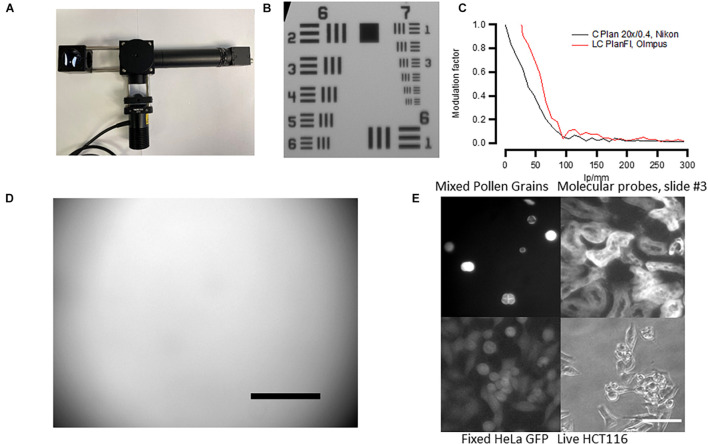
Fluorescent microscope’s performance. **(A)** Assembled microscope and the autofocusing system. See Supplementary Material for the building instructions. **(B)** Micrograph of the USAF 1951 standard. The microscope can resolve Group 7/Element 6 features (2.19 μm size). **(C)** Modulation transfer function calculated from USAF 1951 standard using Fiji SE_MTF plugin (Mitija et al., 2011) Black trace—cheap Nikon Plan 20× non-infinity-corrected objective. Red trace—more expensive Olympus infinity-corrected microscope (see Supplementary Table 2 for more information). **(D)** Micrograph of green Chroma slides showing mild vignetting. Scale bar, 100 μm. Note that the scale bar only provides an approximate value because non-infinity-corrected objective was used. **(E)** Example of the autofocusing system performance in both white transillumination and fluorescence. Scale bar, 30 μm.

The schematics of the fluorescent microscope are shown in Figures 3C,D, 4A. It is a standard inverted fluorescent microscopy system with the following key features. We use a non-infinity-corrected objective (Nikon Plan 20/0.4 or, alternatively, small aspheric lens, *f* = 2.75, *NA* = 0.64) and a 100-mm camera lens (*D* = 25.4 mm). The GFP cube consists of blue and green Thorlabs filters (MF469-35 and MF525-39) and a dichroic mirror (MD498, Thorlabs). The objective is not attached to the rest of the microscope but is moving separately in a Z-stage (Figure 3D), connected to the main frame *via* an aluminum extrusion. If the size of the sample is an issue, a more expensive infinity-corrected objective can be used instead. In this case, there will be no fluctuations in the estimated size when focusing varies from well to well.

### Automatic Control and Electric Circuits

All motors are operated *via* three CNC shields connected to Arduino Mega *via* a PCB board (Supplementary Figure 3). Shield 1 operates the XY stage, perfusion module, and autofocusing (Arduino pins 5–12 and enabling pin 13). Shields 2 and 3 operate eight perfusion pumps (Arduino pins 23–53, odd numbers). In order for the software to have accurate estimates of the position of each axis and pump, endstops are attached to the rail of each axis (Arduino pins 22–44, even numbers). The Gerber file for PCB generation can be downloaded from https://github.com/frescolabs/FrescoM/tree/master/hardware/pcb.

The microscope is operated by two sets of LEDs—transmitting white light for bright-field microscopy and excitation blue (488 nm) light for fluorescent microscopy. Four white LEDs connected in series (Supplementary Figure 2) are then connected to Mosfet IRF520, which, in turn, is connected to a 12-V power supply, ground, and pin 46 of the Arduino. The blue LED is operated using a TTL pulse applied to a Thorlabs LED driver connected to pin 48 of the Arduino.

Additional pins are reserved for the microSD card (Arduino pins 46–52, even numbers), rotary encoder (Arduino pins 2–4), and small LCD display connected to Arduino *via* i2c protocol (Arduino pins A4 and A5). The circuit can be modified to accommodate more CNC shields to increase the overall number of pumps to 22.

### Software

The hardware is operated *via* an Arduino board that receives instructions from a computer *via* serial port. Functionality where commands are sent *via* wifi module or stored in a file in a microSD card are reserved for future versions (all commands are shown in Supplementary Table 1).

The main operating software (Figure 1B) is written in Python 3 and can be downloaded from https://github.com/frescolabs/FrescoM/blob/master/software. The software is very basic, easy to use, and modifiable to fit individual needs. The key functions are as follows:

1. Choose COM port to connect to Arduino (button “Serial”).
2. Move platform forward, backward, left, and right. Two sets of buttons allow for large steps or single-step movements to be made. The step size can be set.
3. Move application manifold and objective up and down.
4. Return to zero position—returns XY platform, the application manifold, and Z-focus into the start position.
5. Set top-right and set bottom-right positions of the multiwell plate.
6. Switch ON and OFF white and blue LEDs.
7. Increase or decrease camera exposure.
8. Move pumps forward and backward. To be used to fill pumps with solutions and cleaning the system after the experiment.
9. Run experiment. Each protocol is implemented as a Python class inherited from a BaseProtocol class (see Supplementary Text for more information) and overriding the self.perform() function. The following classes should be used for running the key functions:

a. FrescoXYZ moves multiwell plate in X and Y, manifold, objective, and syringe pumps.
b. ZCamera operates the key camera functions.
c. ImageStorage saves files generated by the camera.

A few examples of protocol classes are shown in Supplementary Protocols 1–3.

## System Performance

### Optical Resolution

To define the microscope’s (Figure 4A) resolution, we used the USAF 1951 test chart (Figure 4B). For 20× objective and under white illumination, we can observe the sixth set of elements in group 7 giving us a resolution of at least 228 line pairs per millimeter. The size of one pixel was 0.365 μm for a 20× objective. These numbers give only approximate values as the platform employs non-infinity-corrected objectives, and during focusing, the distance between the objective and the camera lens may vary. We have also calculated the slant edge modulation transfer function (MTF) of the designed microscope using USAF 1951 (Figure 4C) and found that the resolution of our system was lower than that in similar open-source microscopes (Merces et al., 2021). This was probably due to the camera resolution as higher-quality objectives only slightly improved the MTF (Figure 4C, red). We therefore advise the users to consider more expensive, infinity-corrected objectives with higher NA, and higher-resolution cameras if high-resolution imaging is required for their experiments.

The fluorescent illumination showed mild vignetting. This was tested by using a fluorescent slide with auto-fluorescence evenly distributed across the slide (Chroma, #92001). The result of such test is shown in Figure 4D—the illumination is well-centered but there is less illumination on the sides of the micrograph, a sign of vignetting. This result has to be taken into account when measuring fluorescent reporters and comparing brightness in individual cells across the same micrograph.

Because we aimed at creating a cheap system, we used non-infinity-corrected objectives that do not allow for correct size estimation and are lower quality. However, in some applications, it is critical to have high-quality objectives with infinity correction. We therefore tested the system’s performance using a range of different objectives (Supplementary Table 2). Three different infinity-corrected objectives (EC Plan Neofluar, Zeiss; LC Plan Fl, Olympus; and C Plan L, Leika) produced similar resolution to our objective but were considerably better in terms of vignetting and the quality of fluorescent images.

### System Vibration and Robustness

To test whether there is any significant vibration in the system, we took 50 images, 1 image every 1 s without making the system perform any other tasks (Supplementary Protocol 1). We then found the key points in each image using the SIFT algorithm (Supplementary Figure 6) and calculated transformation between the key points using the RANSAC algorithm for each pair of photos (2,500 data points altogether). Based on this information, we calculated the translation vector and found that the average difference between individual frames was 1.8 pixels (0.475 μm) and the maximum difference was 6 pixels (2.19 μm). When the application manifold is moving, a small vibration in the system was detected during the first second of movement (Supplementary Video 4). In this case, the average distance between individual frames was 4.3 pixels (1.56 μm) and the maximal error was 13.8 pixels (5 μm). Autofocusing also produced a small displacement (13 ± 8 pixels, 4.745 μm on average).

To test how well the system moves from well to well, we repeated the same analysis, but in this case, we moved the multiwell plate from A1 position to H8 and back (Supplementary Protocol 2). The plate always went to the same well and approximately the same field of view; however, the position slightly varied from trial to trial. On average, the offset was 295 pixels (107 μm), which corresponds to 20% of the field of view and 0.1% of the total distance moved. Thus, parallel imaging of multiple well plates requires bringing the plate position to exactly the same position using additional image analysis and programming and/or image registration after the experiment is completed. When calculated separately, the displacement on *x*- and *y*-axes was 159 and 153 pixels (58 and 55 μm), respectively.

### Autofocusing

The designed autofocusing system allows for automatic focusing when moving from well to well (Supplementary Video 1). The autofocusing algorithm is based on measuring the image sharpness and consists of the following steps.

**Step 1**. Move the objective to zero position.

**Step 2**. Move the objective to a position defined by the user (stored in the ZCamera: auto_focus_anchor variable in ZCamera.py file).

**Step 3**. Move the objective up from the position defined in the step 2 moving by five motor steps 20 times (these values are stored in the ZCamera:auto_focus_delta_number_of_jumps and ZCamera:one_jump, respectively). At each focal plane, the algorithm calculates the focus measure, a value that defines how sharp the image is at this focal plane.

**Step 4**. Position the objective 10 steps lower than the position where maximum sharpness is achieved.

**Step 5**. Repeat step 3 with smaller steps (two motor steps).

We have implemented and tested four different measures of sharpness described in Bueno-Ibarra et al. (2005): (1) Tenengrad (TENG)—a sum of the square of X and Y magnitudes of a Sobel operator of an image; (2) MLOG—a maximum value of the Laplacian operator of an image; (3) LAPM—average of the Laplacian operator of an image; and (4) variance of a Laplacian operator of an image. We found that TENG is the most robust for both wide field and fluorescent images (Supplementary Figure 4). The result of the autofocusing performed on different samples using TENG is shown in Figure 4E.

### Perfusion System

**FIGURE 5:**
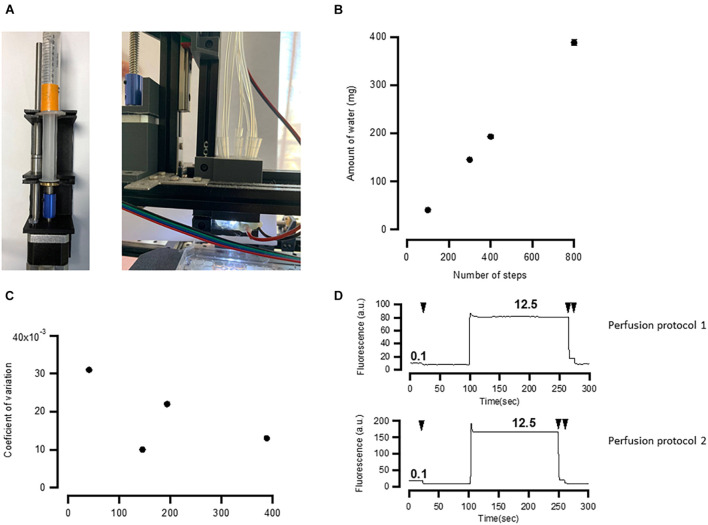
Application manifold performance. **(A)** Assembled pump and solution application manifold. **(B)** Relationship between number of steps and the amount of water ejected by the 10-ml syringe. The relationship is linear. **(C)** Random error of the pumps calculated expressed as coefficient of variation of 10 measurements. **(D)** Application and washout of fluorescent solution (Lucifer Yellow) recorded by the fluorescent microscope. See details in the main text. Washouts are shown by arrows. Both perfusion protocols exhibit ∼99% washout. Numbers indicate the concentration of fluorescent compound in μg/ml. Two back-to-back washouts were more effective in both protocols, as revealed by the further decrease in fluorescence after the second washout.

To test how well the perfusion system (Figures 3E, 5A) exchanges solutions in individual wells, we have conducted two series of tests. First, we have estimated the linearity and errors of the volume released by the syringe pumps as a function of the number of steps moved by the pump’s stepper motor (Figures 5B,C). We then used fluorescent solution to estimate how well individual wells are perfused by the developed system.

To test the robustness of the syringe pumps, we applied different steps of the stepper motor driving the syringe pump and weighed the amount of water that is ejected in each individual session. This was repeated 10 times for the same number of steps and for a 10-ml syringe. The results of this experiment are shown in Figures 5B,C. The amount of the ejected water linearly depends on the number of steps (Figure 5B), and the error, calculated as a coefficient of variance of 10 measurements, is between 1 and 3%. This is approximately 3–10 times as large as required by ISO 86552.

How well the perfusion system exchanges liquids in a well was tested using two different solutions. One solution contained deionized water, while the other contained 12.5 μg/ml Lucifer Yellow dissolved in deionized water. These two solutions were sequentially applied while taking images by the fluorescent microscope under blue light illumination. To give a rough estimation of how well the solution is washed out, we first applied 0.1 μg/ml of Lucifer Yellow and then washed it out observing a small decrease in fluorescence. This suggests that the sensitivity of the camera is sufficient enough to detect a change from 0.1 μg/ml of Lucifer Yellow to 0 (Figure 5D). If fluorescence after washout drops to the level of 0.1 μg/ml, then it means that at least 99% of the fluorescent solution is washed out. To test this, we used two perfusion protocols. In one protocol (Perfusion Protocol 1), we applied the fluorescent solution while sucking the excess using a peristaltic pump connected to a tip located a few millimeters above the other tips. The result of this experiment is shown in Figure 5D, top. In the second test, we used Perfusion Protocol 2, in which we positioned one of the applying tips lower than the others, sucked the solution *via* this tip using a syringe pump, and then applied another solution (Figure 3B, bottom). Both methods yielded a similar degree of washout (>99%) after two back-to-back washouts with Perfusion Protocol 2 using smaller volumes but providing less control over the liquid level. Both ways of perfusion have their advantages and can be used in different experimental paradigms. For instance, when one needs quick application of agonist using functional imaging, Perfusion Protocol 1 may be preferable as it offers better control over liquid level. On the other hand, methods, such as immunolabeling, particularly using expensive antibodies or other chemicals, may benefit from Perfusion Protocol 2.

## Discussion

There is a high demand for designing affordable and flexible tools for high-scale generation of biological data. Here, we report a combination of hardware and software that allows for up to several hundred cell biology experiments performed simultaneously and automatically. The framework here and the recently developed OpenTrons-based framework (Ouyang et al., 2021) allow for simultaneous solution handling and microscopy experiments. Below, we discuss applicability of the developed experimental platform and possible ways for its future improvement.

### Importance of Automation of Biological Experiments

Automation of biological experiments is important for two main reasons. First, a large number of data points are required when using modern methods of analysis, such as machine learning and deep learning. A good convolutional neural network algorithm typically requires in the region of 10,000–100,000 data points (O’Mahony et al., 2019). Considering that there are only a few thousands of cells in a field of view, the same experiments need to be reproduced 10–100 times. This number increases significantly if the experimental goal is to optimize conditions for biological experiments.

Second, there is a growing discussion on reproducibility of biological data (Ioannidis, 2005; Pusztai et al., 2013; Freedman et al., 2015; Miura and Norrelykke, 2021). This is particularly crucial when the results have direct translational applications and can affect future expensive clinical trials. The reproducibility can be improved when the experiments are standardized and when experimentation and data analysis are performed automatically to avoid human errors. The reproducibility of experiments can be further improved when performed on different sources (different cell types, cells with different genetic backgrounds, etc.), further highlighting the necessity of automation of biological experiments.

### Applicability of the Developed Experimental Platform

#### High-Throughput Screening Experiments

Expansion of the compound libraries (Root et al., 2003; Cases et al., 2005; Rickardson et al., 2006) provided a valuable tool to search for new chemicals affecting biological functions. For example, drugs affecting physiological signaling pathways (e.g., GPCRs) can be assessed by calcium imaging (Berridge, 2001; Bootman et al., 2002; Berridge et al., 2003) or any other forms of functional imaging with a single-cell resolution. However, this requires a large number of experiments, which is laborious. The framework developed here easily allows for automatic labeling of cells with fluorescent labels and reporters (Figures 3D–F), application of different agonists (Figures 3B,G), and measurements of calcium signaling (Figure 3G) in a large number of wells.

#### Optimization Experiments

Many biological experiments require optimization of treatment conditions. For example, differentiation of stem cells requires treatment with a large number of growth factors and morphogens at different times and with different dynamics (Kim et al., 2002; Panchision and McKay, 2002). The framework developed here allows for experiments, such as this to be performed in an automatic manner. The outcome of an experiment can then be automatically tested using one of two methods: labeling of cells with synthetic dyes (Figure 3E) and antibodies against appropriate surface markers or a functional experiment (e.g., neurons can be detected by calcium imaging and application of potassium chloride or neurotransmitters).

#### Large-Scale Characterization of Cells Derived From Individual Patients

Substitution of standard cell lines with cells recently derived from individual patients is now becoming increasingly important (Rominiyi et al., 2019; Stringer et al., 2019). The experimental framework developed here would allow a large number of cell lines derived from individual experiments to be tested in a standardized way.

#### Routine Cell Biology Procedures

The developed experimental platform will also be useful in routine lab procedures, such as concentration dependence curves, cell dilutions, and cytotoxicity studies.

#### Data Collection for Deep Learning Model Training

Tasks, such as cell segmentation and classification may be solved by deep learning but they require a large number of cells to be automatically recorded and labeled. The developed platform can be used to image thousands of cells and combine fluorescent and bright-field imaging to perform automatic labeling of cell borders, nuclei, and specific cell types (e.g., differentiated vs. non-differentiated stem cells).

#### Education

The developed platform will also be extremely useful for teaching in higher education institutions. It has a potential to assist in teaching the basics of optics, bright light and fluorescence microscopy, microfluidics, building scientific equipment, and python coding for experimental automation and data analysis.

### System’s Limitations and How to Address Them

#### Increasing Optical Resolution

When developing the fluorescent microscope, we aimed at making it as cheap as possible but using high-quality filters and dichroic mirrors to improve the fluorescent signal. Therefore, we decided to use a cheap camera with a relatively small sensor size and non-infinity-corrected objectives that can readily be found on any microscope. As a result, the optical system is not idealistic in two major respects.

First, a mild vignetting is observed, which results in unequal excitation of the fluorescent sample (Figure 4C). This can be improved by extending the beam through substituting the 100-mm tube lens with one that has a longer focal distance. This could also allow the use of a camera with a larger sensor and larger number of pixels, which in turn will improve the spatial resolution of the system.

The second disadvantage is that we have used a non-infinity-corrected objective that results in variable distances between the objective and the tube lens. This should lead to a small error in size estimation and lower resolution as defined by the MTF (Figure 4). This can be addressed by using infinity-corrected objective. In this case, the camera should be positioned precisely in the focus of the tube lens, which is not required in the current configuration (Supplementary Figure 5).

#### Increasing the Robustness of the Mechanical System

The precision of the developed system can be further improved by using better mechanical parts. For example, substitution of the standard T8 lead screws with a T8-2 for the *x*- and *y*-axes will decrease the step size. Using 8-mm nuts with a spring will also likely improve the precision as it will reduce the vibration in the system. Step size can also be decreased through using and configuring a DRV8825 motor driver instead of the A4988 used here. These measures will increase the precision of the system but will decrease its speed.

Any rotational movements arising from the fact that both X and Y lead screws are located aside of the plate can be reduced by adding a second stepper motor for each axis located on the other side of the plate. However, this will reduce the number of stepper motors that can otherwise be used for syringe pumps.

#### Improving Perfusion

Single chamber perfusion achieves ∼99% of the solution washout (Figure 5D), which is sufficient for most possible applications. However, if the experimental design requires washout of the active compound, this may pose a problem as concentration curves often span for two to three orders of magnitudes (Communi et al., 1996, 1999; Hur et al., 2004), and therefore, even after the washout, cells can be stimulated with the active compound. Therefore, it is recommended to do several washouts sequentially to achieve better solution exchange (Figure 5B). High-precision glass syringes (e.g., Hamilton) may also improve the robustness of solution application.

#### Decreasing Price

The price of the system can be decreased by several hundred pounds in the following ways.

1. Substitute Thorlabs LED (£222) with cheaper 460-nm 1-W alternatives (e.g., Bright Blue LED from Future Eden—£1.09).
2. Substitution of the Thorlabs LED driver (£242) with RCD24 driver (£21).
3. Substitution of the GFP filter cube (£577) with a filter cube from an old fluorescent microscope (free).
4. Substitution of FLIR camera (£370) with Raspberry Pi camera (v24, £47).

The substitutions described above will decrease the price of the microscope but will also likely lead to a worsening of the imaging quality and stability. Therefore, they should be applied with caution.

### Further Development

The modular structure of the platform developed here allows for fairly easy future adjustments to fit individual experimental needs. For example:

1. Substitution of the microscope (Figure 3) with a heat block will allow to incorporate PCR capability into the analysis. Other means of detection of the experimental outcome (e.g., mini spectrophotometer) will increase the amount of experiments that can be performed.
2. Currently, the microscope only provides simple wide-field one-color fluorescence. It can be improved by adding some optical sectioning functionality, such as HiLo or light sheet microscopy (OpenSPIM).
3. The number of applied solutions can be increased by redesigning the solution application manifold and the PCB.
4. Cleaning the multi-syringe system can be automated by adding storages for distilled water, alcohol, and waste.
5. Experiments can take a long time to perform and cells may need occasional passaging. An algorithm of detecting confluence and then splitting cells into new wells would allow the outcome of experiments to not be affected by overconfluence. In addition to that, selection of individual cells and transferring them into a new well will allow for generation selection of new clones for generation of new lines using transfection or CRISPR.

## Materials and Methods

### Cell Cultures

SKBR3 and HeLa cells were grown in standard Dulbecco modified Eagle’s media (DMEM) supplemented with 10% fetal bovine serum. Cells were passaged once a week when they reached 90% confluence.

### Fluorescent Labeling

Fluo4-AM and Oregon Green BAPTA-1 AM (Thermo Fisher Scientific) were dissolved in DMSO (50 μg per 50 μl). Ten microliters of stock solution and 3 μl of pluronic acid were dissolved in 10 ml, added to the cells. Cells were then incubated at 37°C for 45 min. Imaging procedure is described in the main text.

### Production of Recombinant Herceptin Antibody

The recombinant Herceptin antibody was generated by transfecting a 10-cm^2^ dish of HEK cells with 5 μg of heavy chain (pFUSEss-CHIg-hG1) and 5 μg of light chain (pFUSE2ss-cLIg-hK) plasmids in complex with 50 μg of PEI. Three days post-transfection, the media was collected from the cells and frozen.

## Data Availability Statement

The hardware and software published in work are freely available under Apache 2.0 license. All hardware, firmware, software, and Python protocols are available at https://github.com/frescolabs/FrescoM. Spinnaker license agreement can be downloaded at https://www.flir.co.uk/globalassets/support/iis/knowledge-base/flirspinnaker-sdk-eula-2018.pdf.

## Author Contributions

AN and PK: conceptualization, mechanics and electronics design, mechanics and electronics implementation, and software design. AN and AC: fluorescent microscope design. PK: software implementation. PK, AC, AN, and AP: writing—original draft. AN and JZ: biological experiments. OS and AP: generation of herceptin antibodies. All authors contributed to the article and approved the submitted version.

## Conflict of Interest

The authors declare that the research was conducted in the absence of any commercial or financial relationships that could be construed as a potential conflict of interest.

## Publisher’s Note

All claims expressed in this article are solely those of the authors and do not necessarily represent those of their affiliated organizations, or those of the publisher, the editors and the reviewers. Any product that may be evaluated in this article, or claim that may be made by its manufacturer, is not guaranteed or endorsed by the publisher.
