# Supplementary material for: An Open-Source Framework for Automated High-Throughput Cell Biology Experiments
Source: Front Cell Dev Biol. 2021 Sep 24;9:697584. doi: 10.3389/fcell.2021.697584 (PMC8498207; doi:10.3389/fcell.2021.697584)
Supplement: Supplementary file 1 [file Data_Sheet_1.docx]

Supplementary Materials

**An open-source framework for automated high-throughput cell biology experiments**

Pavel Katunin, Jianbo Zhou, Ola M. Shehata, Andrew A. Peden,^,^ Ashley Cadby, Anton Nikolaev*

**This PDF file includes:**

Supplementary Text

Figs. S1-S4

Table S1

Links to Suplementary Videos S1-S3

**Other Supplementary Materials for this manuscript include the following:**

Supplementary Files S1-S3 – examples of used protocol classes

**SUPPLEMENTARY TEXT**

This file contains assembly instructions, software user guide and supplementary figures referenced in the main text.

**Bill of materials**

The overall cost of the system mechanical part of the system is £622 which includes 8 syringe pumps. This can be reduced if smaller number of pumps is needed.

| **Name or part number** | **Price (£)** | **Link** |
| --- | --- | --- |
| **Optical parts from Thorlabs and the camera** | | |
| CM1-G01 | 136.31 | <https://www.thorlabs.com/newgrouppage9.cfm?objectgroup_id=1670&pn=CCM1-G01> |
| ER2-P4 | 17.91 | <https://www.thorlabs.com/thorproduct.cfm?partnumber=ER2-P>4 |
| C6W | 50.72 | <https://www.thorlabs.com/thorproduct.cfm?partnumber=C6W#ad-image-0> |
| FFM1 | 46.46 | <https://www.thorlabs.com/thorproduct.cfm?partnumber=FFM1#ad-image->0 |
| B3C/M | 19.81 | <https://www.thorlabs.com/thorproduct.cfm?partnumber=B3C/M> |
| MD498 | 177.74 | <https://www.thorlabs.com/newgrouppage9.cfm?objectgroup_id=2990&pn=MD498#2993> |
| MF525-39 | 193.16 |  |
| MF469-35 | 193.16 |  |
| CP33/M - 2 parts | 12.67 | <https://www.thorlabs.com/thorproduct.cfm?partnumber=CP33/M> |
| SM1L05 – 3 parts | 9.73 | <https://www.thorlabs.com/newgrouppage9.cfm?objectgroup_id=3307&pn=SM1L05#3388> |
| SM1L35 | 28.96 |  |
| M470L4 | 222.38 | <https://www.thorlabs.com/newgrouppage9.cfm?objectgroup_id=2692&pn=M470L4#10802> |
| LEDD1B | 242.66 | <https://www.thorlabs.com/thorproduct.cfm?partnumber=LEDD1B> |
| KPS101 | 26.52 | <https://www.thorlabs.com/newgrouppage9.cfm?objectgroup_id=8861> |
| SM1T2 | 16.16 | <https://www.thorlabs.com/thorproduct.cfm?partnumber=SM1T2> |
| SM1A9 | 14.97 | <https://www.thorlabs.com/newgrouppage9.cfm?objectgroup_id=1524&pn=SM1A9#3235> |
| Flir camera  BFS-U3-16S2 | 370 | <http://softwareservices.flir.com/BFS-U3-16S2/latest/Model/spec.html> |
| Camera to USB cable DCV/ACC-01-2300 | 6.07 | <https://uk.rs-online.com/web/p/usb-cables/9015064/?cm_mmc=UK-PLA-DS3A-_-google-_-CSS_UK_EN_Cables_%26_Wires_Whoop-_-USB+Cables_Whoop-_-9015064&matchtype=&pla-353144551868&gclid=CjwKCAjwxo6IBhBKEiwAXSYBs8utKG6ZqN4y55yN1ZpEdF6pDqWSi4CkC7ZEFokxCkxJLJaIl-2fdhoCBwQQAvD_BwE&gclsrc=aw.ds> |
| Non-infinity corrected objective, 20x | Free | Use any non-infinity corrected objective. For better image quality use more expensive infinity-corrected objectives. |
| 100 mm lens  AC254-100-A | 60.87 | <https://www.thorlabs.com/thorproduct.cfm?partnumber=AC254-100-A> |
| 20 mm lens | 14.69 | <https://www.thorlabs.com/thorproduct.cfm?partnumber=ACL2520U> |
| **Total** | 1892.99 | See main text to find out how to decrease the price of the microscope by several hundred pounds. |
| **Mechanical parts and electronics** | | |
| PLA, 2.85, black | 39.00 | <https://ultimaker.com/resellers> |
| MakerbeamXL 15x15 starter kit | 107.81 | <https://www.makerbeam.com/makerbeamxl-regular-starter-kit-black.html> |
| Nema17 steppermotors (x12) | 60.00 | e.g. <https://www.amazon.co.uk/SIMAX3D-stepper-42x38mm-Creality-extruder/dp/B08FFM6LRL/ref=sr_1_2_sspa?dchild=1&keywords=nema%2B17%2Bstepper&qid=1628597598&sr=8-2-spons&spLa=ZW5jcnlwdGVkUXVhbGlmaWVyPUFGV0NKMzU2VjRaMzMmZW5jcnlwdGVkSWQ9QTA1Nzk0NzUzU0k2TkdINFVDMVhBJmVuY3J5cHRlZEFkSWQ9QTA1NzI2NjgzUlIwWUtQRkdTTDhMJndpZGdldE5hbWU9c3BfYXRmJmFjdGlvbj1jbGlja1JlZGlyZWN0JmRvTm90TG9nQ2xpY2s9dHJ1ZQ&th=1> |
| Linear guide rails. 400mm | 14.60 | <https://www.banggood.com/Machifit-MGN12-100-1000mm-Linear-Rail-Guide-with-MGN12H-Linear-Sliding-Guide-Block-CNC-Parts-p-1156260.html?utm_source=googleshopping&utm_medium=cpc_organic&gmcCountry=GB&utm_content=minha&utm_campaign=minha-gb-en-pc&currency=GBP&cur_warehouse=UK&createTmp=1&ID=515971&utm_source=googleshopping&utm_medium=cpc_bgs&utm_content=sxxx&utm_campaign=ssc-gb-all-enw-0624-20lp&ad_id=444236244939&gclid=CjwKCAjwx8iIBhBwEiwA2quaq7ZtlFPknwnD2LVqzKeuMwS6fPIBfFjJNrXH7P5LY2AygPH7hLc8PxoCYbQQAvD_BwE> |
| Linear guide rails. 250mm (x3) | 32.85 |  |
| Linear guide rails. 100mm (x2) | 22.02 |  |
| Screw rods with nut (x9) | 9.99 x 9 | <https://www.amazon.co.uk/Screw-250mm-Printer-Threaded-Linear/dp/B06WP1WZSY/ref=sr_1_14?dchild=1&keywords=3d+printer+screw+250+mm&qid=1584744051&sr=8-14> |
| Screw rods with nut. 400 mm. | 12.49 | <https://www.amazon.co.uk/Screw-250mm-Printer-Threaded-Linear/dp/B06WRN1DQS/ref=sr_1_14?dchild=1&keywords=3d%2Bprinter%2Bscrew%2B250%2Bmm&qid=1584744051&sr=8-14&th=1> |
| Nema17 motor driver (x12) | 15.98 for 10 | <https://www.amazon.co.uk/HALJIA-Stepstick-Stepper-Driver-Printer/dp/B0793K9KF8/ref=dp_prsubs_2?pd_rd_i=B0793K9KF8&psc=1> |
| Mini limit switch (x12) | 10.79 | <https://www.amazon.co.uk/KingYH-Momentary-Instrument-Communications-Electronic/dp/B07YDFBS5X/ref=sr_1_33?crid=2AEO1981JUHD&dchild=1&keywords=arduino+limit+switch&qid=1584744462&sprefix=arduino+limit+swit%2Caps%2C160&sr=8-33> |
| Arduino mega | 35.70 | <https://www.amazon.co.uk/ARDUINO-MEGA-2560-REV3-A000067/dp/B0046AMGW0/ref=sr_1_3?dchild=1&keywords=Arduino+Mega&qid=1584746050&sr=8-3> |
| Box of screws and nuts | 4.67 | <https://www.amazon.co.uk/CONNEX-DP8500055-Machine-Screw-Nut/dp/B00B22VHPC/ref=sr_1_5?keywords=screw+and+nuts&qid=1584746128&sr=8-5> |
| CNC Shields (x3) | 17.97 | <https://www.amazon.co.uk/ARCELI-Engraver-Printer-Expansion-Arduino/dp/B07CQRPFFW/ref=sr_1_7?dchild=1&keywords=CNC+shield&qid=1593538726&sr=8-7> |
| LM8UU linear bearing (x8) | 9.22 | <https://www.amazon.co.uk/Vzer-Linear-Bearings-Printer-RepRap-Silver/dp/B01LXHHAAB/ref=sxts_rp_s1_0?cv_ct_cx=LM8uu&dchild=1&keywords=LM8uu&pd_rd_i=B01LXHHAAB&pd_rd_r=3a324528-86b9-4350-9f75-1810b97c4cac&pd_rd_w=gcNhx&pd_rd_wg=miY9Q&pf_rd_p=a0c48f7c-59fe-409f-82e9-de7d87ff898f&pf_rd_r=F0GCGABSAFEREC1TG71H&psc=1&qid=1627660244&sr=1-1-1890b328-3a40-4864-baa0-a8eddba1bf6a> |
| 8mm Rods | 9.69 x 4 | <https://www.amazon.co.uk/sourcing-map-Lathe-Round-Length/dp/B07KT3MXV6/ref=sr_1_5?dchild=1&keywords=8mm+rod&qid=1593539702&sr=8-5> |
| IRF520 MOSFET | 2.9 | <https://www.amazon.co.uk/HCMODU0083-IRF520-MOSFET-Driver-Module/dp/B012ATHAN8> |
| White LED (box) | 4.99 | <https://www.amazon.co.uk/gp/product/B07FCR8W2H/ref=ppx_yo_dt_b_search_asin_title?ie=UTF8&psc=1> |
| Bench Power supply | 58.89 | e.g. <https://www.amazon.co.uk/3-Digital-Adjustable-Switching-Regulated-Alligator/dp/B07L3K5952?ref_=ast_sto_dp> |
| 2.54 pins, both male and female and jumpers | 12.99 | e.g. <https://www.amazon.co.uk/WayinTop-Connectors-Assortment-Computer-Straight/dp/B0868LJDW1/ref=bmx_dp_u9kcebpn_6/261-6038049-7356365?pd_rd_w=JvWy0&pf_rd_p=eb113e0f-8c39-4b39-8cfa-62247fe4355e&pf_rd_r=8N4XZKW6NWTZNBBWNBVF&pd_rd_r=e970dc46-fd9f-4ad0-bb7b-f872b5fff3ed&pd_rd_wg=TsuUo&pd_rd_i=B0868LJDW1&psc=1> |
| Collection of wires | 6.99 | <https://www.amazon.co.uk/Elegoo-120pcs-Multicolored-Breadboard-arduino-colorful/dp/B01EV70C78/ref=pd_bxgy_img_1/261-6038049-7356365?pd_rd_w=Dehe4&pf_rd_p=c7ea61ca-7168-47e3-9c8b-d84748f5b23c&pf_rd_r=H5FJ3RAKKBZZZKNPTW5R&pd_rd_r=b30b8b88-fd55-4bba-8fd7-f032d6b80e3f&pd_rd_wg=usvxm&pd_rd_i=B01EV70C78&psc=1> |
| Male BNC connector | 5.99 | <https://www.amazon.co.uk/Kagni-Crimp-Connectors-RG59-cable/dp/B07RSMSNKW/ref=sr_1_5?dchild=1&keywords=BNC+connector&qid=1627660859&s=electronics&sr=1-5> |
| Male/Female BNC cable | 7.49 | <https://www.amazon.co.uk/sourcing-map-Bulkhead-Coaxial-Extension/dp/B07VGKLSS7/ref=sr_1_3?dchild=1&keywords=BNC+cable+male+female&qid=1627660985&s=electronics&sr=1-3> |
| Endstops | 6.99 | <https://www.amazon.co.uk/GTIWUNG-Mechanical-Endstop-Printer-Arduino/dp/B07VWRS24K/ref=sr_1_1_sspa?dchild=1&keywords=endstop+limit+switch&qid=1631273820&sr=8-1-spons&psc=1&spLa=ZW5jcnlwdGVkUXVhbGlmaWVyPUEzNE9ISVhFWDhVOVRJJmVuY3J5cHRlZElkPUEwODEzNjExMzYwWUZPNDdERjcxSiZlbmNyeXB0ZWRBZElkPUExMDQ0MDg1MUI2WUw4N1A2NldCTCZ3aWRnZXROYW1lPXNwX2F0ZiZhY3Rpb249Y2xpY2tSZWRpcmVjdCZkb05vdExvZ0NsaWNrPXRydWU>= |
| **Total** | 629.00 |  |

**Assembly instructions**

Building the system requires some time (approximately 2 days), concentration, calmness and basic soldering skills. Please contact Anton Nikolaev ([a.nikolaev@sheffield.ac.uk](mailto:a.nikolaev@sheffield.ac.uk)) if you have any problems with the assembly.

MakerbeamXL extrusions are connected to each other using aluminium L- and T-shaped brackets. Each bracket is screwed by 5 M3 screws. Think in advance where nuts have to be placed as forgetting a nut may require disassembly of some parts. Below we provide schematic instruction for building the hardware and the electric circuits. The positions of L- and T- brackets are not shown but they can be put anywhere as long as they properly bind extrusions together. Figures below show how to assemble the platform. Black arrows show where you need to insert the nuts **prior to frame assembly.**

Step 1. 3D print all parts required for the hardware. All STL files can be found at <https://github.com/frescolabs/FrescoM/tree/master/hardware/print> We used Ultimaker 3 printer, PLA filament and 0.4 mm nozzle. The filling was 20% and one layer was 0.15 mm.

Generate PCB (the scheme and/or Gerber file can be downloaded from <https://github.com/frescolabs/FrescoM/tree/master/hardware/pcb>).

Step 2. Main frame and the X-axis.

Assemble the main frame, attach X-motor extrusion, X-motor holder and X-axis endstop holder as shown in figures above. Screw the endstop to the holder using M3 screws. Attach MGH12 rails. Notice how extrusions connect with each other (red arrows).


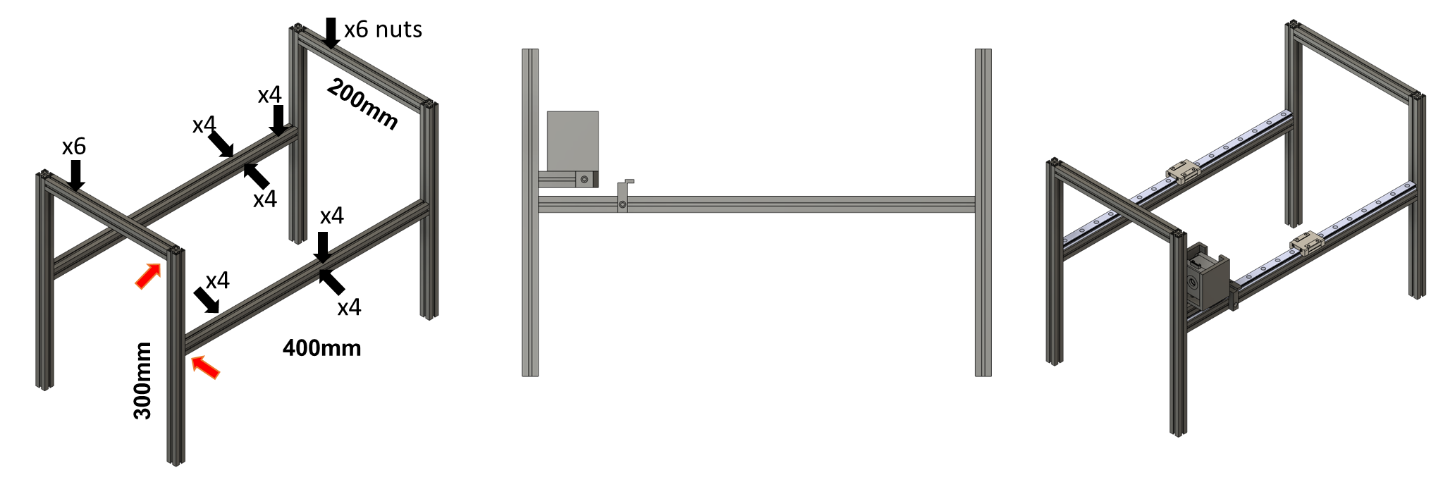


**NB! Remember to insert M3 nuts to the top of lower horizontal extrusions, at least 4 nuts to each side of the lower horizontal extrusions and 4 screws to the inner side of each upper horizontal extrusions before assembling the frame!**

**NB! To assemble 400mm extrusions connect 300mm and 100mm extrusions together.**

Step 3. Y-axis assembly


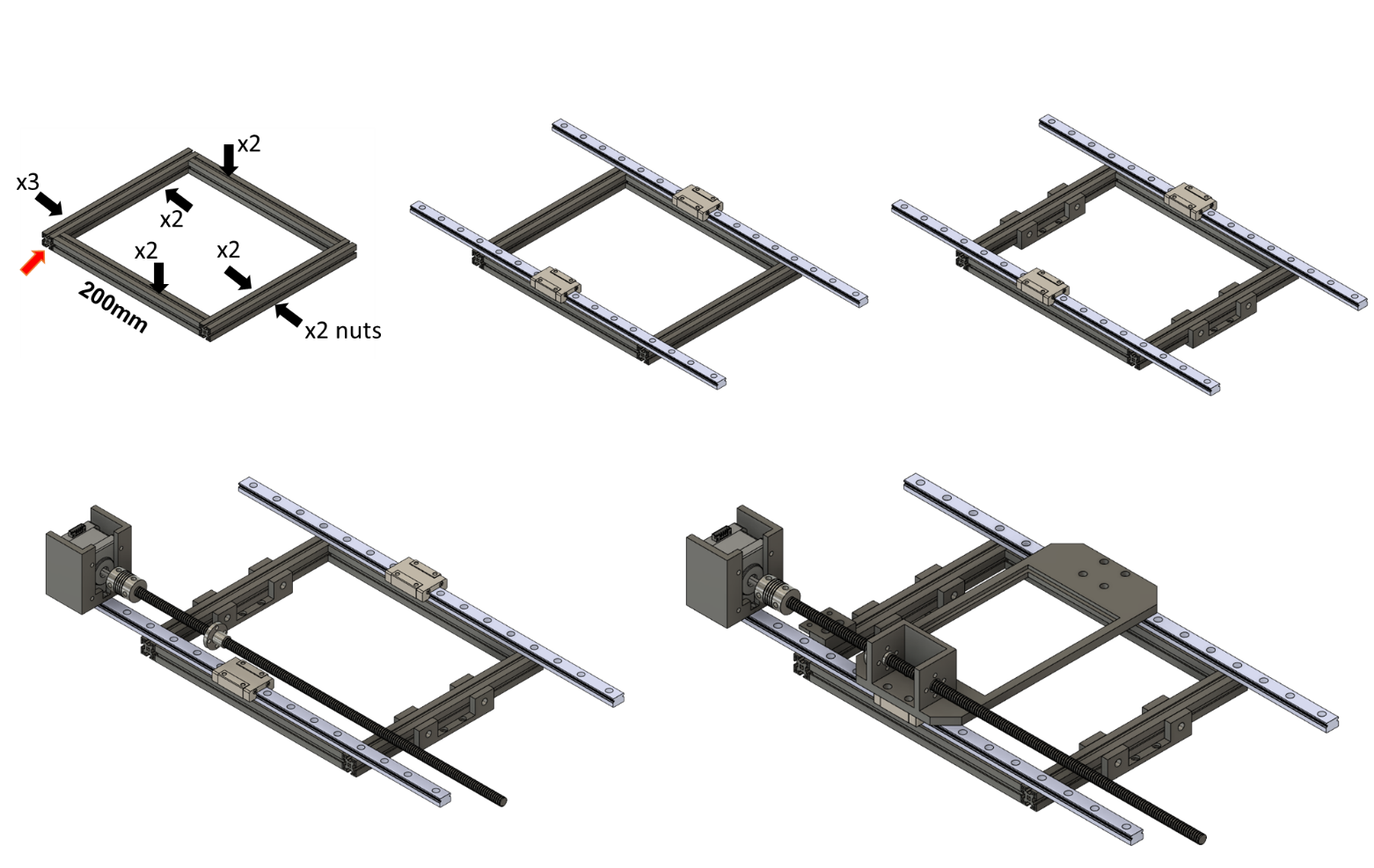


Assemble the Y-axis as shown above. Bind the main frame extrusions using L-shapes positioned below the frame. Attach one endstop to the endstop holder. Notice how extrusions are connected with each other (red arrow). **NB! Add nuts to each hidden extrusion side.**

Step 4. Perfusion manifold assembly.


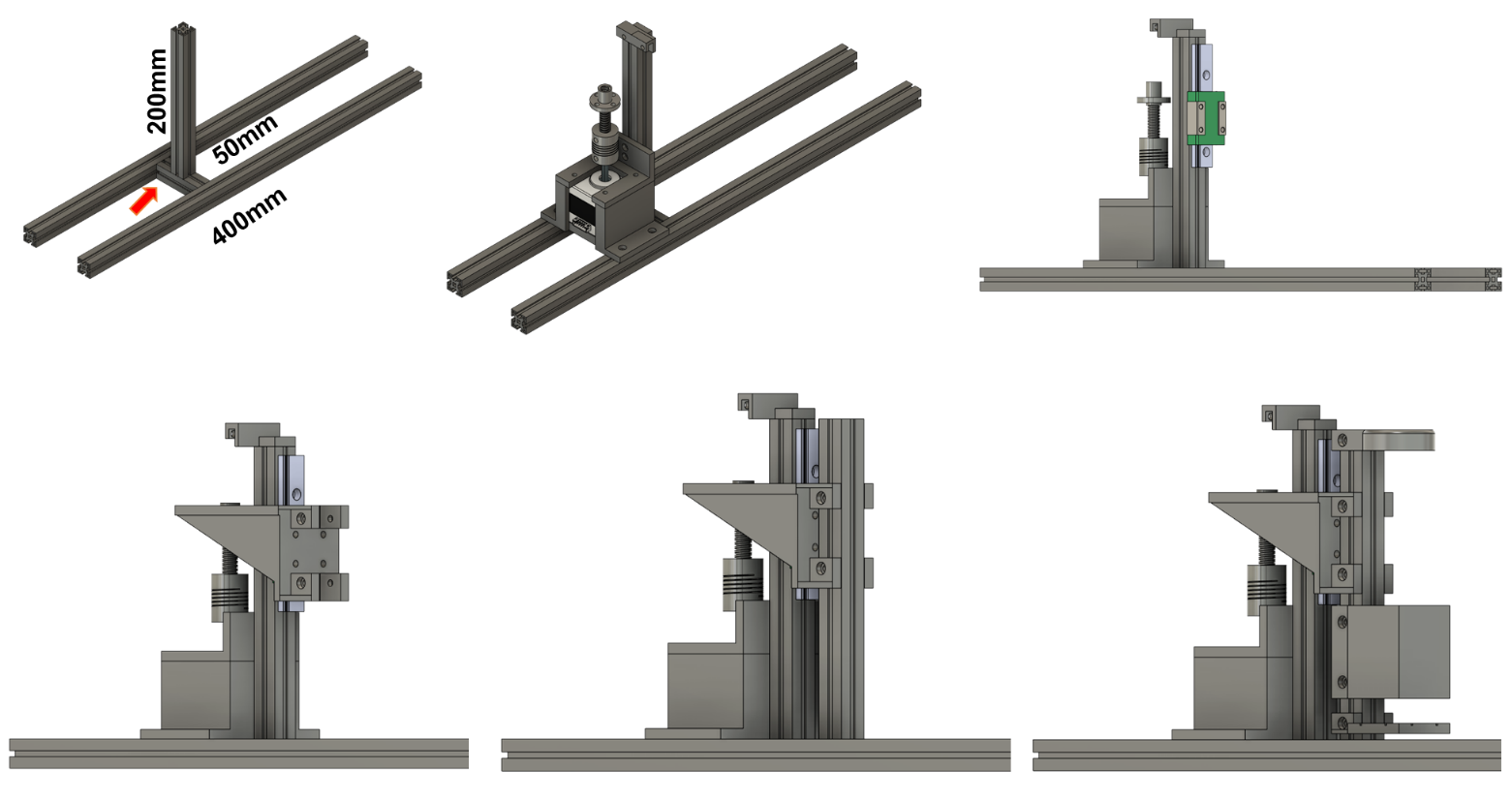


Assemble the perfusion manifold as shown above. Notice how extrusions are connected with each other (red arrows).

**NB! To assemble 400mm extrusions connect 300mm and 100mm extrusions together.**

Attach the endstop to the endstop holder (top of the vertical extrusion). Before mounting the LED holder at the bottom, insert 4 white LEDs, solder them to be connected in sequence and solder unconnected electrodes to a pair of long wires (see Figure below). **NB! Connect all LEDs in series by soldering short pin to long pin of the next LED.** Solder a red wire to longer pin and black wire to shorter pin.


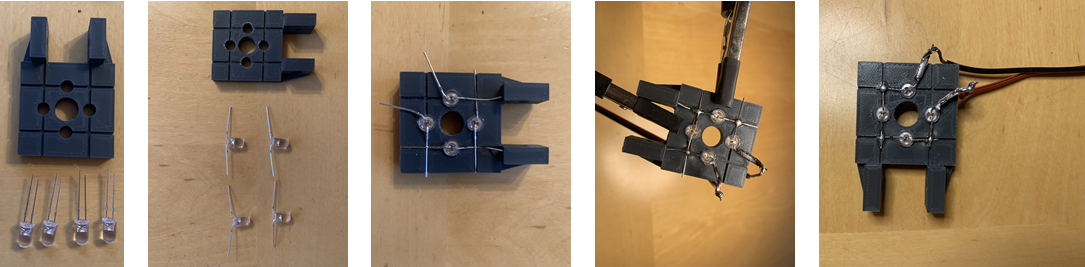


Step 5. Mini microscope assembly

Follow instructions on page 15 to build a small fluorescent microscope using Thorlabs details. The cost of the current configuration is £2000. This can be reduced by £450 if a cheaper LED and constant current driver is used.

**NB! This microscope uses non-infinity corrected objective. If you good size estimation you will need to use infinity corrected objective and a better tube lens.**

Step 6. Focusing system assembly

**
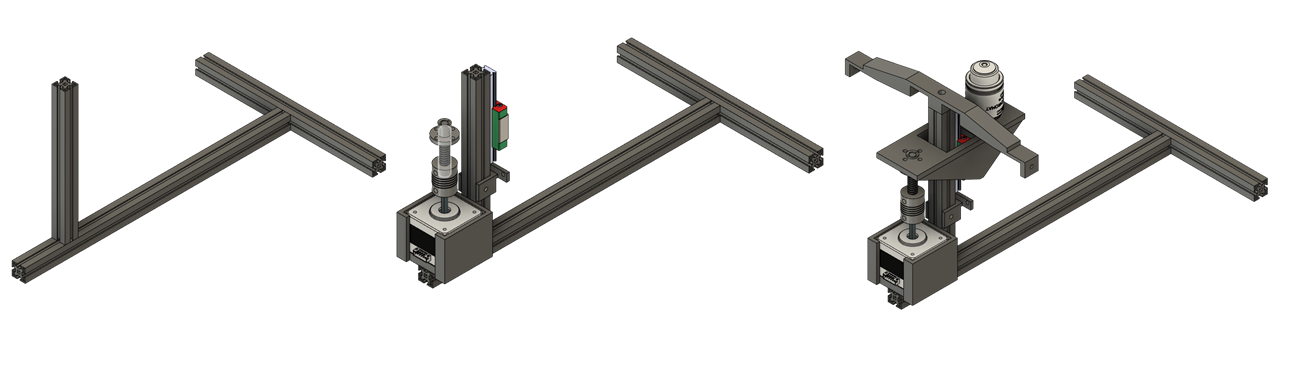
**

Assemble the focusing system as shown above. Attach endstop to the endstop holder.

Step 7. Assemble the microscope (see detailed instruction below) and connect everything together as shown below.

**
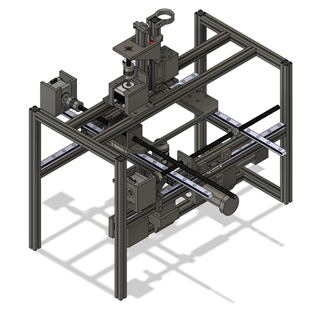
**

Step 8. Connect syringe pumps and/or peristaltic pumps via tubings and gel loading tips as shown below (see also Supplementary Figure S1).


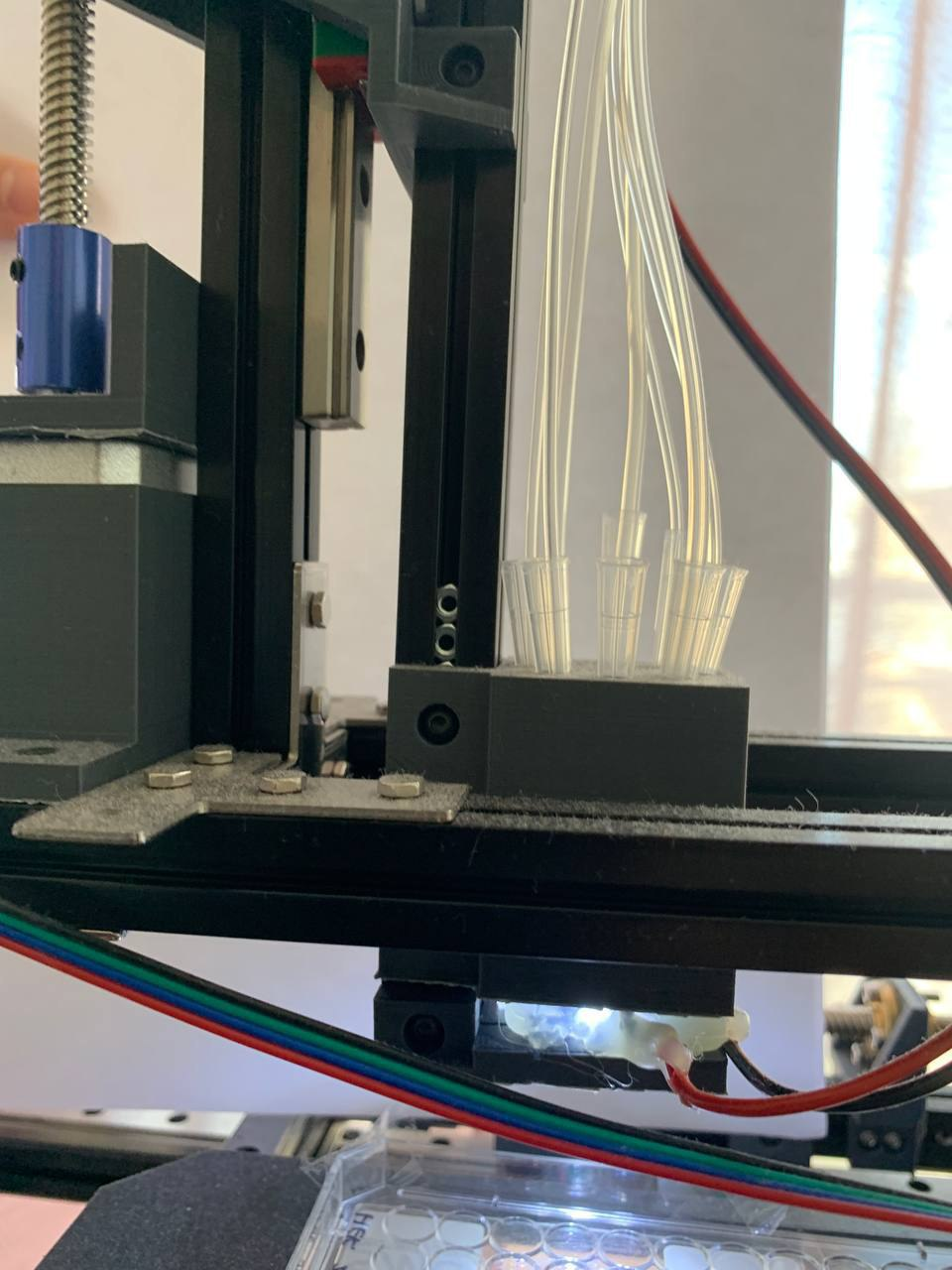


**
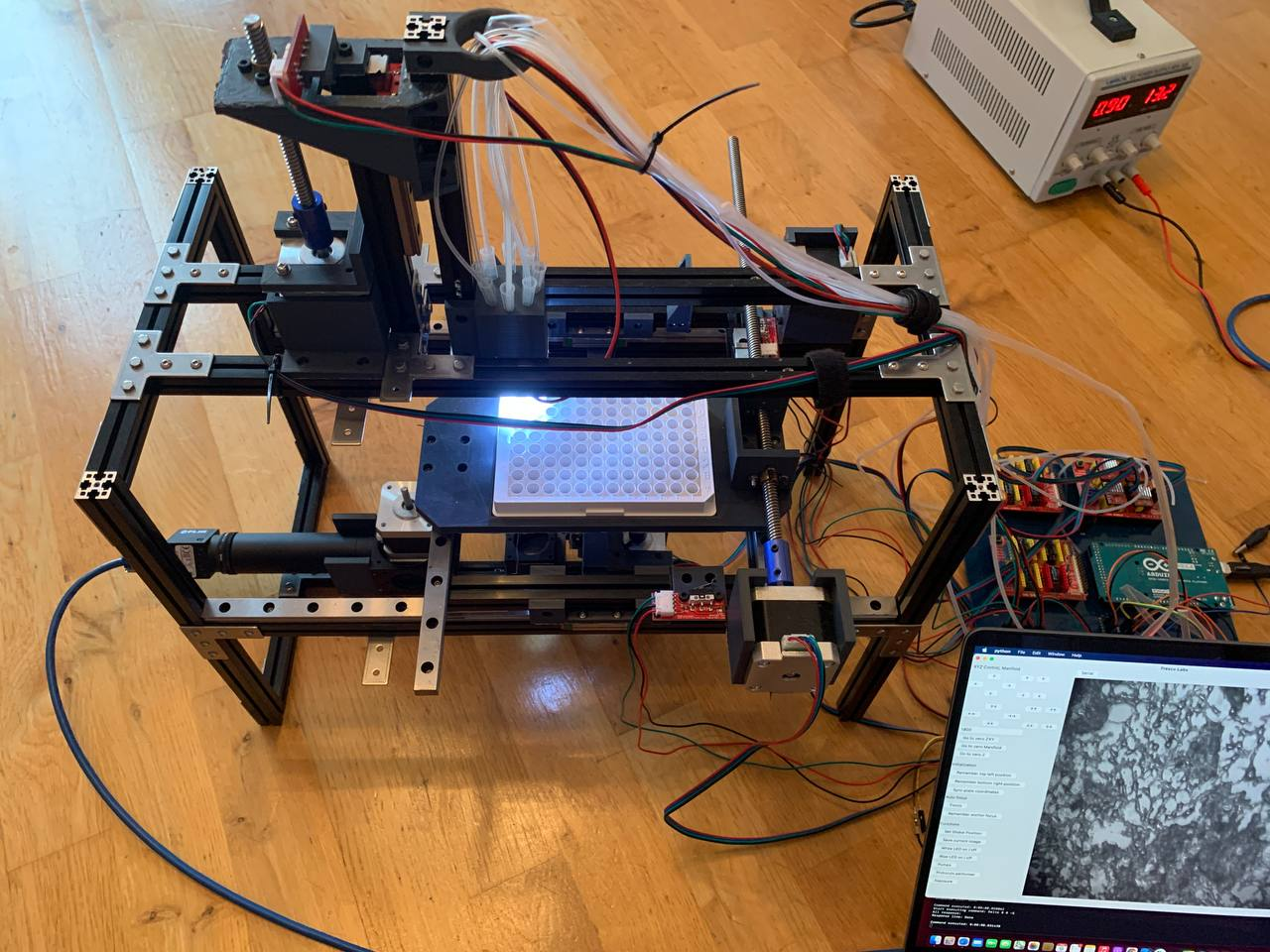
**

Step 9. Electric circuit

We provide all files that can be used to order PCB. All pins need to be soldered by yourself. However, some companies provide this service if you are not willing to solder parts by yourself.

1. Order PCB from preferred supplier (e.g. EasyEDA)
2. Optional: solder all pin slots to the PCB
3. Insert Arduino Mega and all three CNC shields.
4. Connect CNC shields with jumpers as shown in figure below and then insert A4988 drivers.
5. Connect 12V to each CNC shield and to the PCB as shown on figure above.
6. Connect each endstop as shown on figure below. First connect endstops for X-, Y-, autofocus- and perfusion manifold in this order. The rest of the endstops are optional.
7. Connect white LEDs as shown in figure below
8. Connect blue LED driver as shown in figure above. Connect the other side to Thorlabs LED driver.

|  |
| --- |
|  |

**
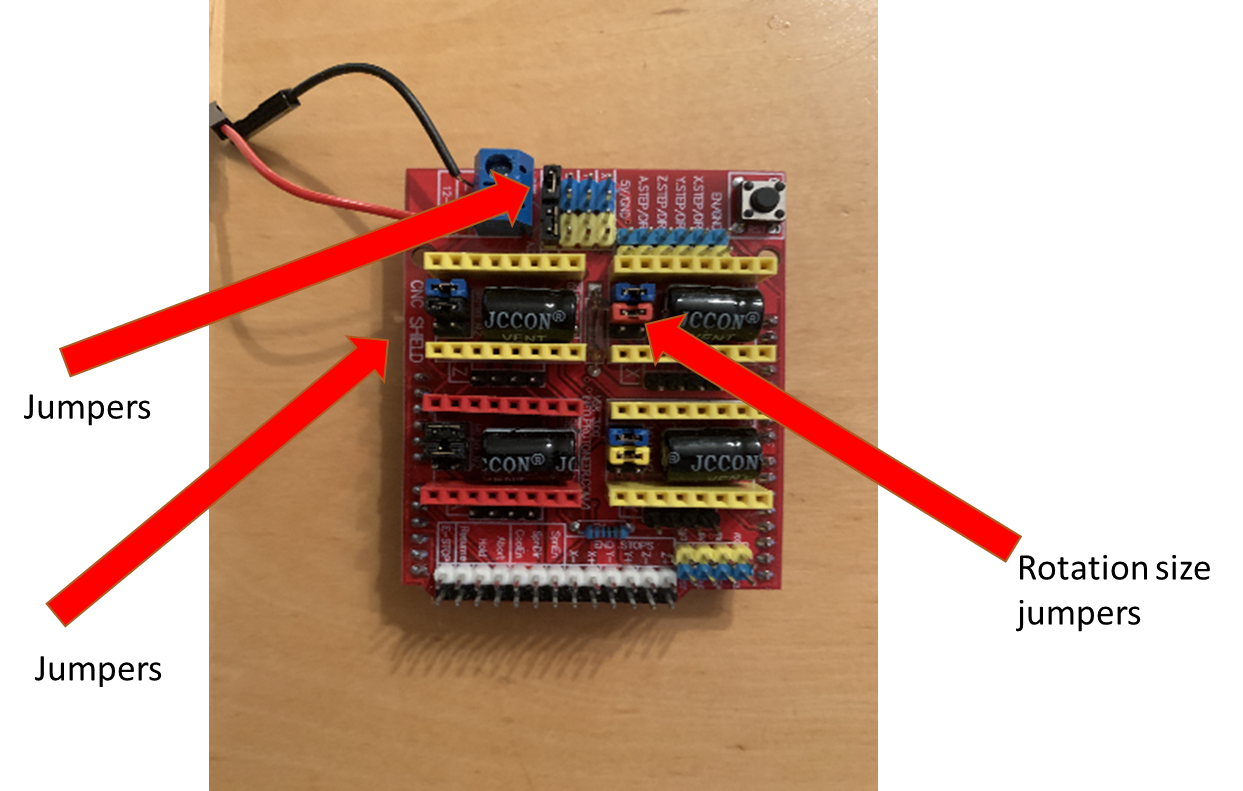
**

**
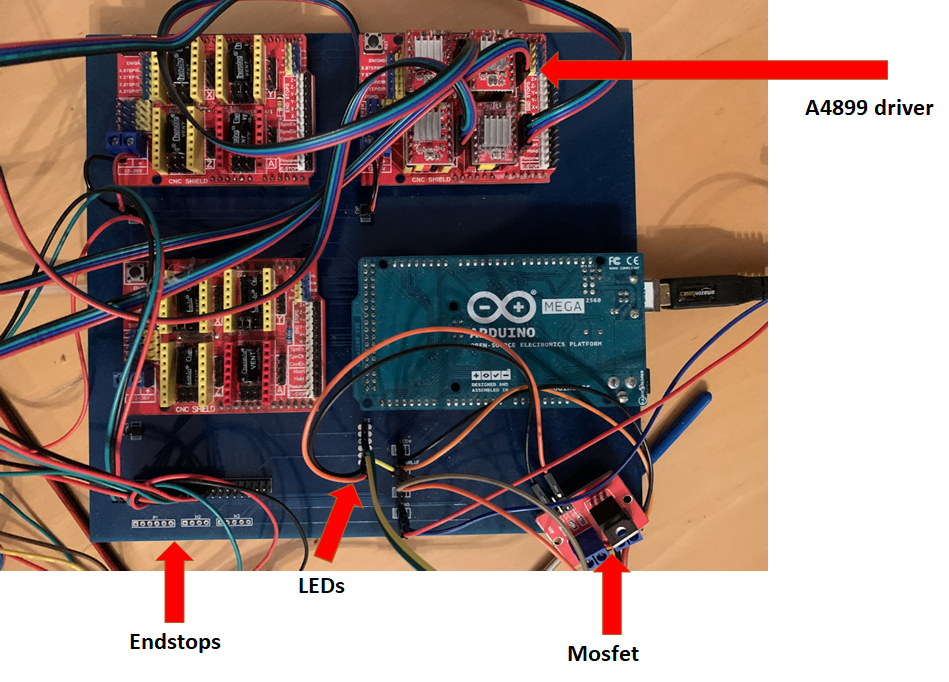
**

**The key PCB connections**

The entire PCB board with soldered pins is shown on figure below:


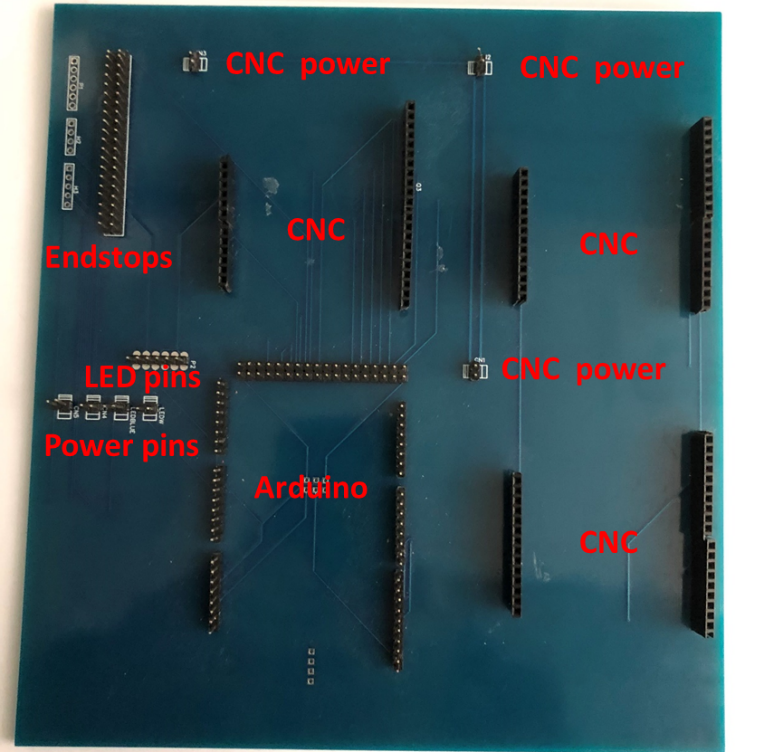


a. Endstops. Figure below shows how to connect the 4 main endstops to the PCB board. Use the following order of endstops: X-axis (most left), Y-axis, autofocusing and application manifold (rightest).


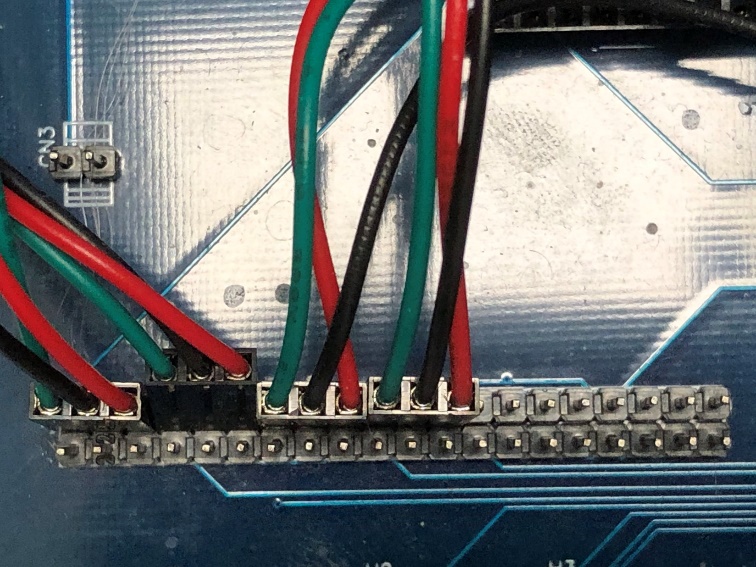


b. Power. Figure below shows the polarity of the power pins:


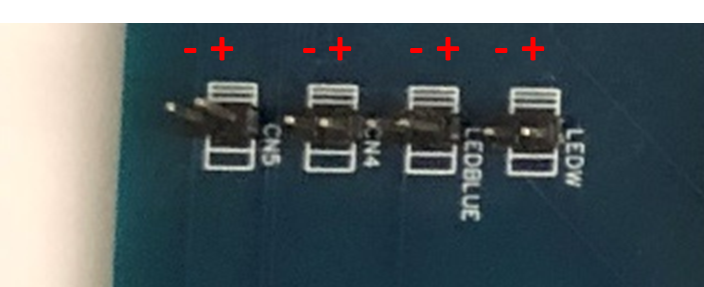


Connect the power supply to CN5 pins using wires with 2.54 pins on one side:


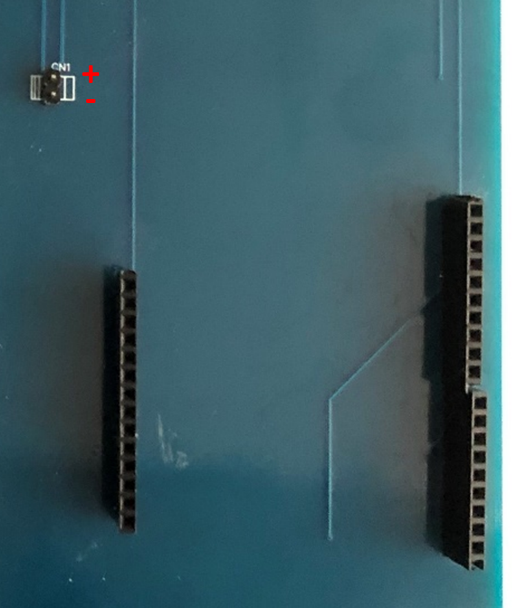

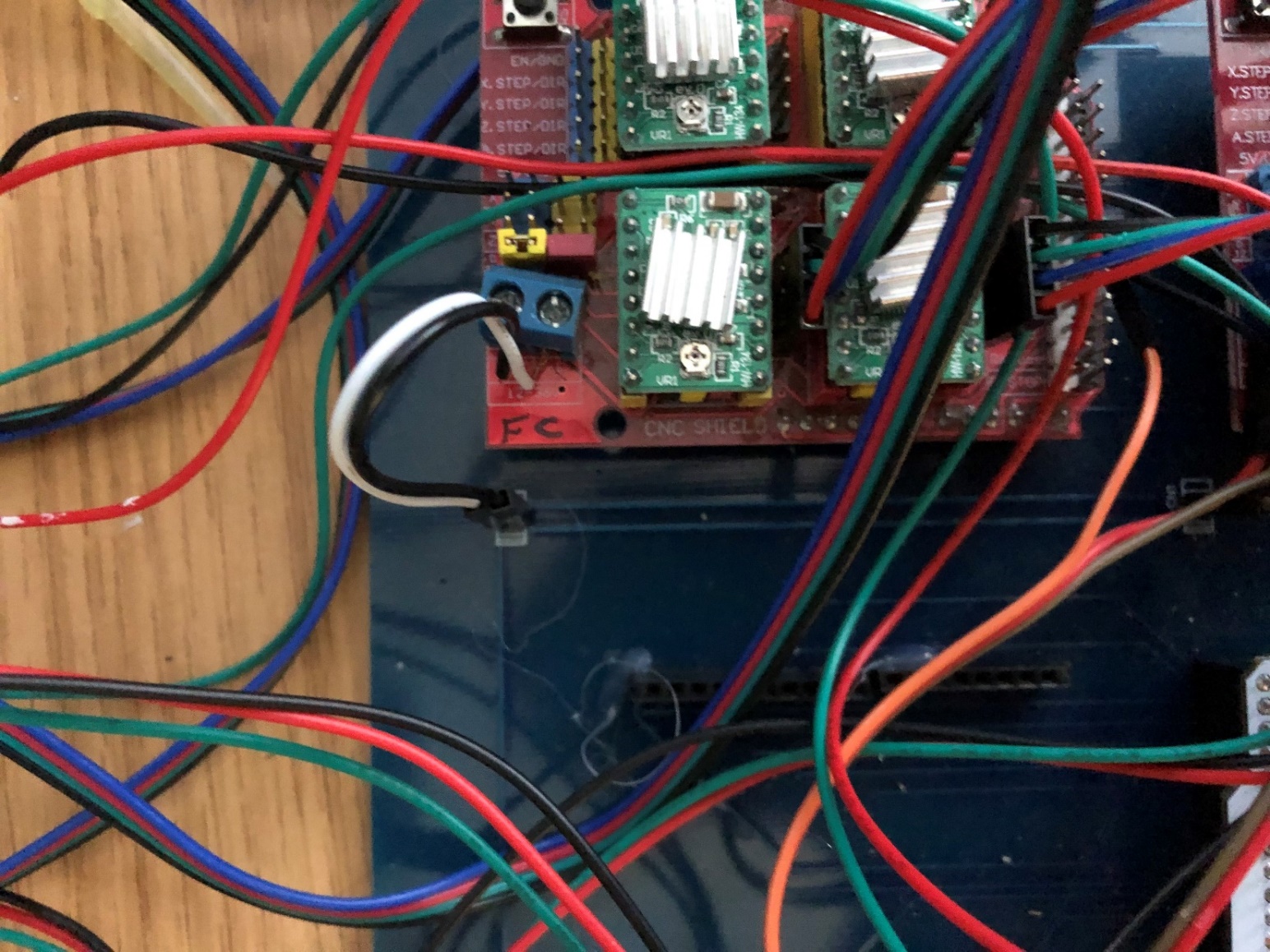


c. LEDs. White and blue LED pins are shown on the figure below:

**
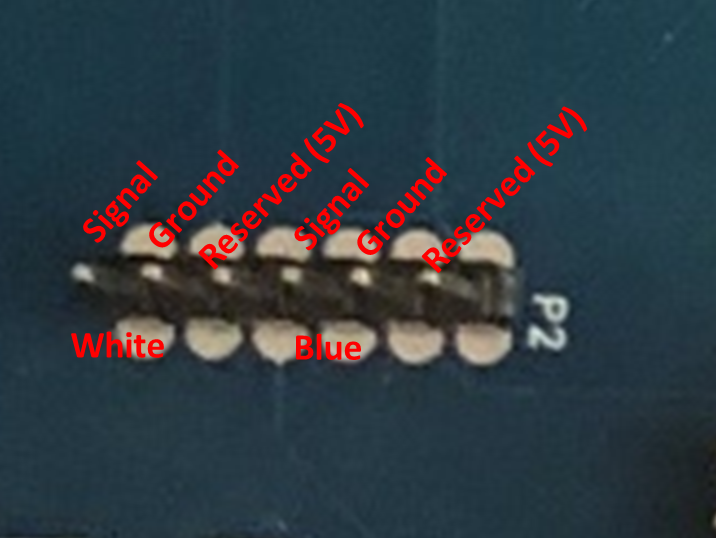
**

Connect the blue LED signal and ground pins (the most left pins on the graph below) to BNC cable and connect it to a T-cube LED driver (Thorlabs, LEDD18). Connect the white signal and ground to a MOSFET as shown below:

**
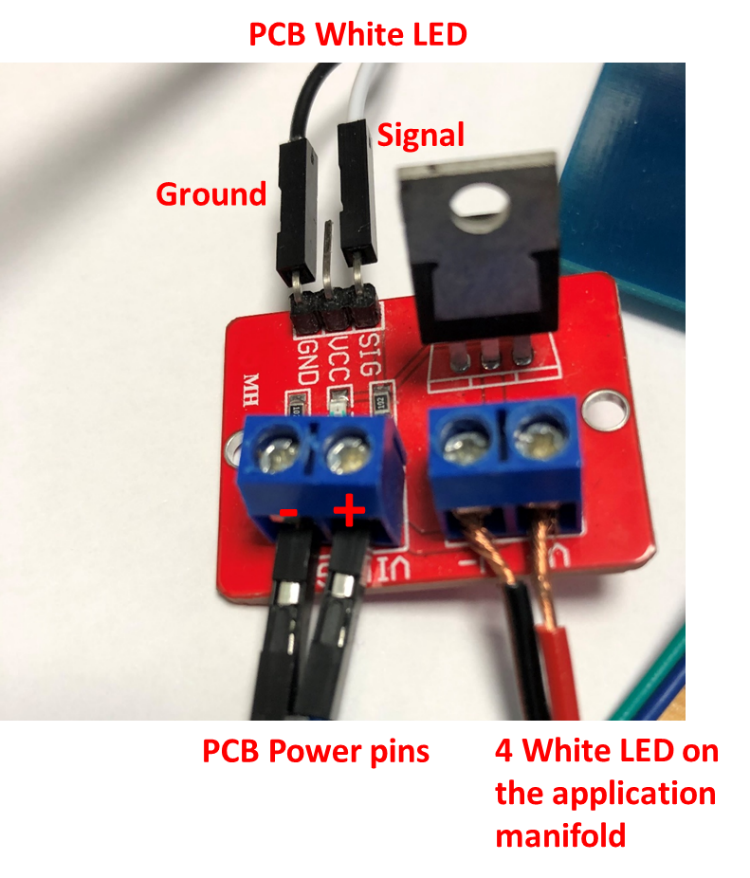
**

**Microscope building instructions**

**NB! If you plan to use an infinity-corrected objective, it is critical that the camera sensor is precisely in focus of the tube lense. To achieve this, rotate the camera around SM1T2 coupler until distant image (e.g. clouds or houses far away) are in focus (Supplementary Figure 4).**

1. Thorlabs optical and mechanical parts and Flir camera are shown on the figure below.

**
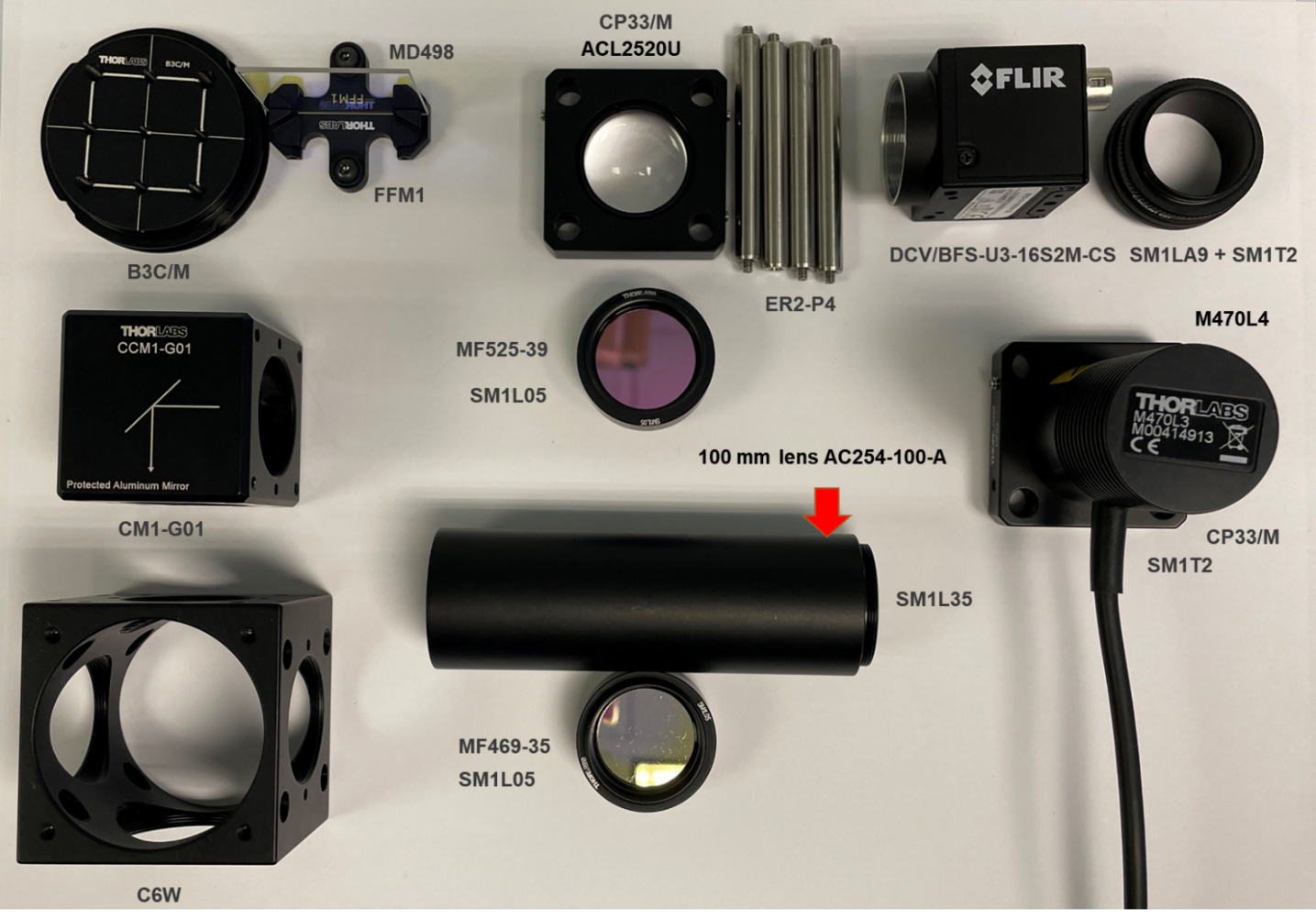
**

2. Assemble the mirror

**
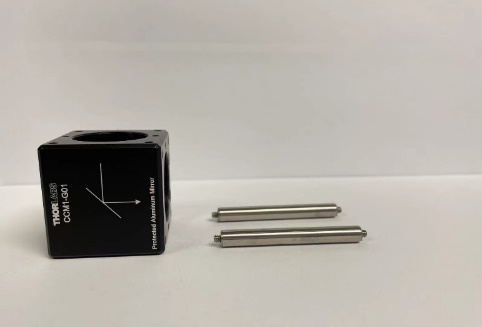

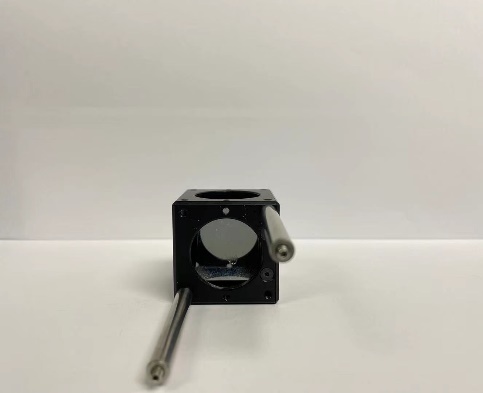
**

3. Assemble the dichroic mirror cube

**
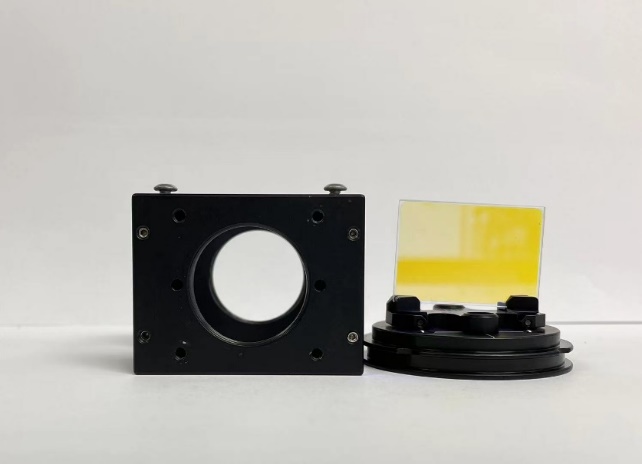

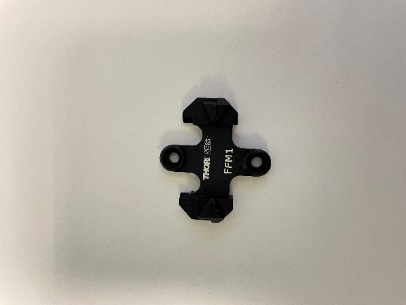

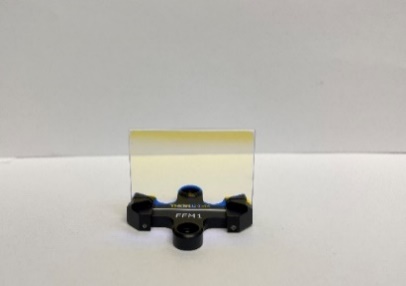

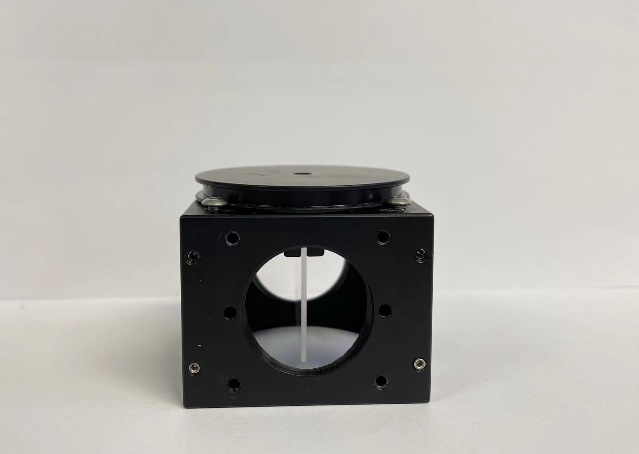
**

4. Add the blue filter mounted in SM105 tube

**
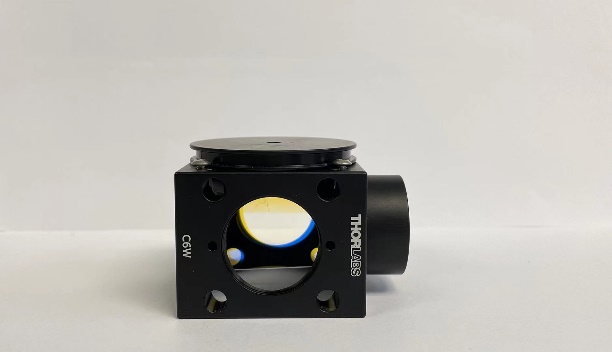
**

5. Add condenser lens mounted on a CP33/M cage plate


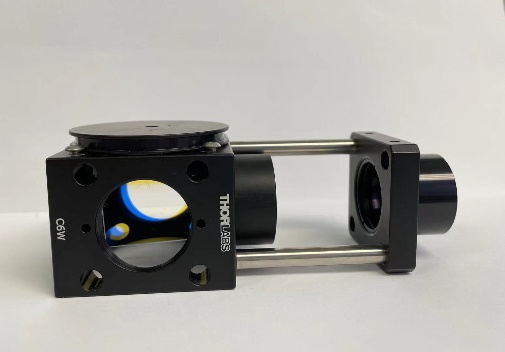


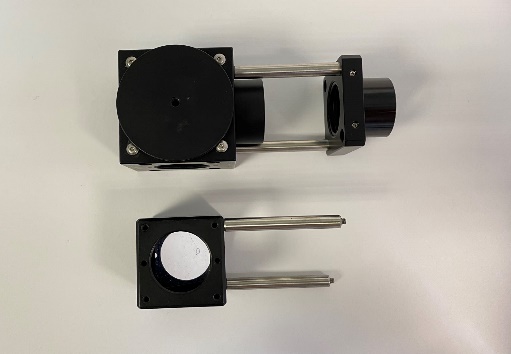
6. Connect the mirror


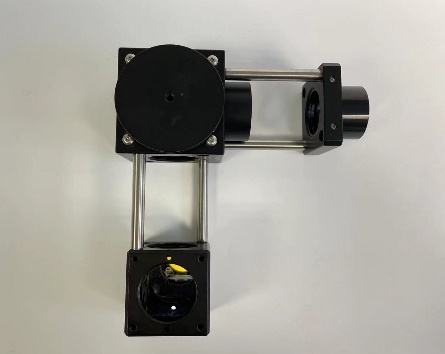


7. Mount green filter, tube lens and camera on SM1L35 with 100mm tube lens positioned at the end of the tube (red arrow).


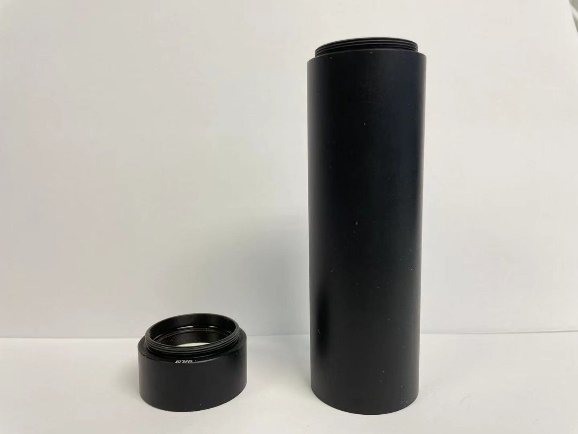

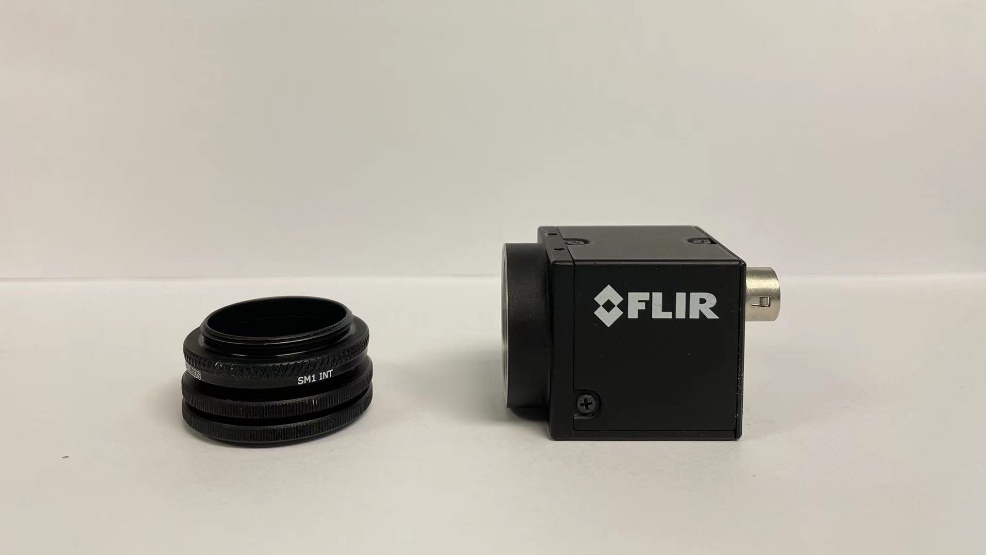

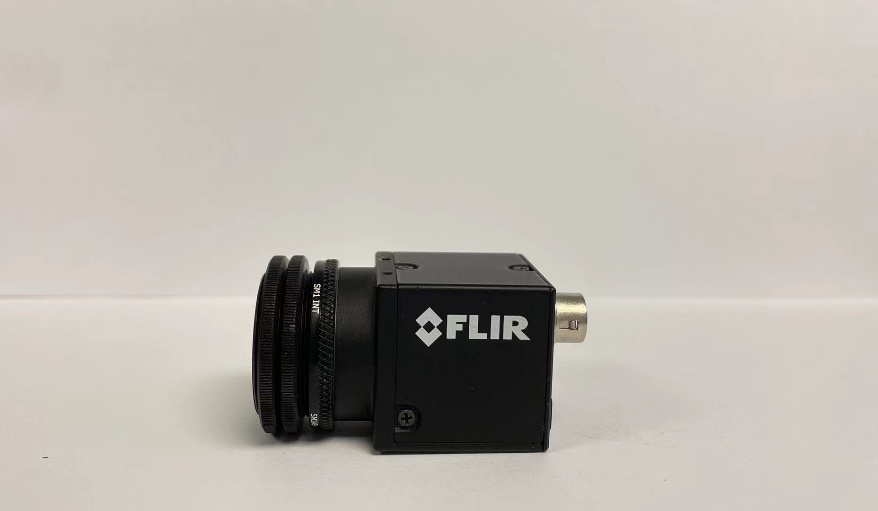

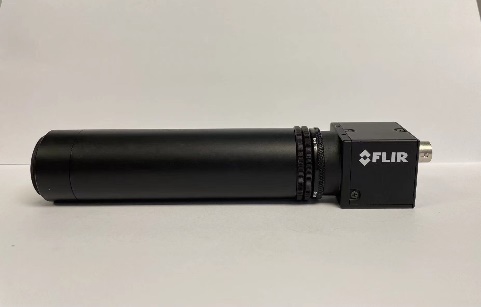

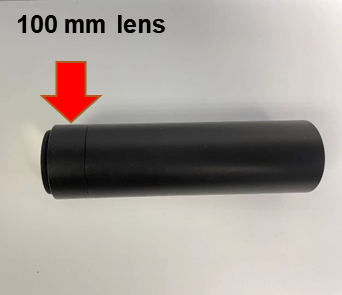


8. Assemble the rest of the microscope


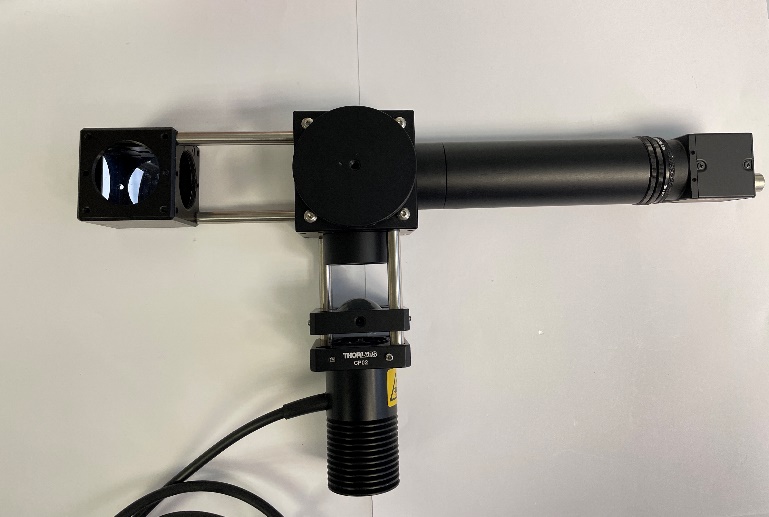

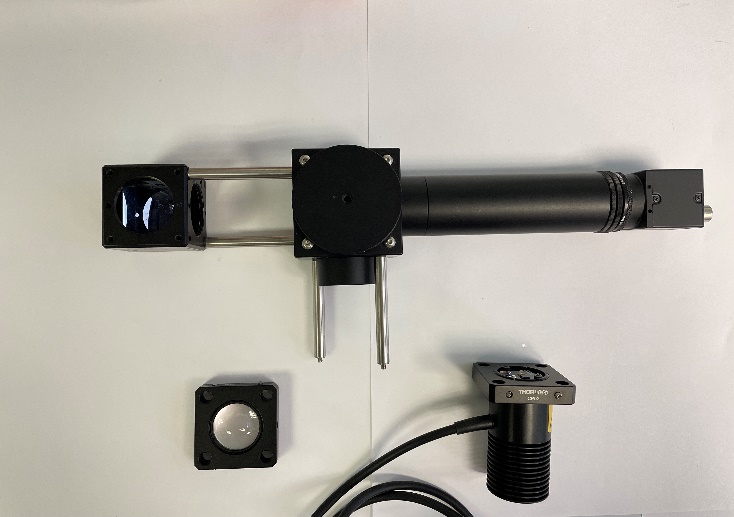


**Firmware**

The hardware is operated via an Arduino board that receives instructions from a computer via serial port. Functionality where commands are sent via wifi module or stored in a file in a microSD card are reserved for future versions. The commands shown in Table 1.

**Protocol programming guide using Python**

The following part describes the main Python classes to be used to generate new protocols. Python has become popular among biologists and therefore, instead of development of complex GUI where new protocols can be set up, we propose programming protocols using Python.

The basics of protocol programming is simple:

1. Generate a new class inherited from BaseProtocol class
2. Override the perform() function
3. Override the constructor
4. Save the new script in software/services/protocols folder. Class name should have the same name as the file but with Snake Case translated to Pascal case. For example, if you name your class AllWellsPhotoProtocol save it in all_wells_photo_protocol.py file.
5. Press “Protocol Performer” button on the main Form, chose the protocol and run it.

Additional steps:

1. Import the following classes:

from services.protocols.base_protocol import BaseProtocol

from services.fresco_xyz import FrescoXYZ

from services.z_camera import ZCamera

from services.images_storage import ImagesStorage

2. Override the constructor in the following way:

def __init__(self,

fresco_xyz: FrescoXYZ,

z_camera: ZCamera,

images_storage: ImagesStorage):

super(AllWellsPhotoProtocol, self).__init__(fresco_xyz=fresco_xyz,

z_camera=z_camera,

images_storage=images_storage)

self.images_storage = images_storage

where AllWellsPhotoProtocol is the name of your class.

3. Use the following functions

**self.fresco_xyz.white_led_switch()** to switch on and off the white LED

**self.fresco_xyz.go_to_zero_manifold()** to move manifold to zero position

**self.z_camera.focus_on_current_object()** to run autofocus

**self.hold_position()** to wait for some time (in seconds)

**self.z_camera.fresco_camera.get_current_image()** to capture an image

**self.fresco_xyz.manifold_delta()** to move manifold certain number of steps

**self.fresco_xyz.delta (x, y, z)** to move XY stage and the focusing system by X, Y or Z steps. If you used hardware and electronics configuration described above move X or Y by 1800 steps to move to the next well.

**self.fresco_xyz.delta_pump ()** to move the given pump by given number of steps

**self.fresco_xyz.blue_led_switch()** to switch on and off the white LED

| **Command** | **Action** |
| --- | --- |
| Zero | Move X and Y to zero position |
| VerticalZero | Move Z to Zero position |
| Position (e.g. Position 1000 1000 1000) | Set position |
| Delta (e.g. Delta 0 100 0) | Move X, Y, Z by the given number of steps |
| RememberTopLeft | Remember top left |
| RememberBottomRight | Remember bottom right |
| GetTopLeftBottomRightCoordinates | Send coordinates for the plate |
| ManifoldZero | Move Manifold to zero position |
| DeltaPump 0 -100 | Move pump # 0 for -100 steps |
| ManifoldDelta | Moves manifold |
| SwitchLedW | Switch on and off top white LED |
| SwitchLedB | Switch on and off blue LED for microscope |
| GetCurrentPosition | Returns current XYZ |

**Supplementary Table 1. List of commands used by Arduino to operate the main hardware.** The Arduino code can be downloaded from https://github.com/frescolabs/FrescoM/blob/master/firmware

| **Objective**  **Key features** | **Price** | **Infinity corrected** | **Chroma slide** | **Chroma slide fluorescence profile** | **USAF 1951** | **Example of a fluorescent**  **image (cells labelled with**  **Fluo-4 calcium dye)** |
| --- | --- | --- | --- | --- | --- | --- |
| Nikon Plan20/0.4  20x | Used: 0 | No | **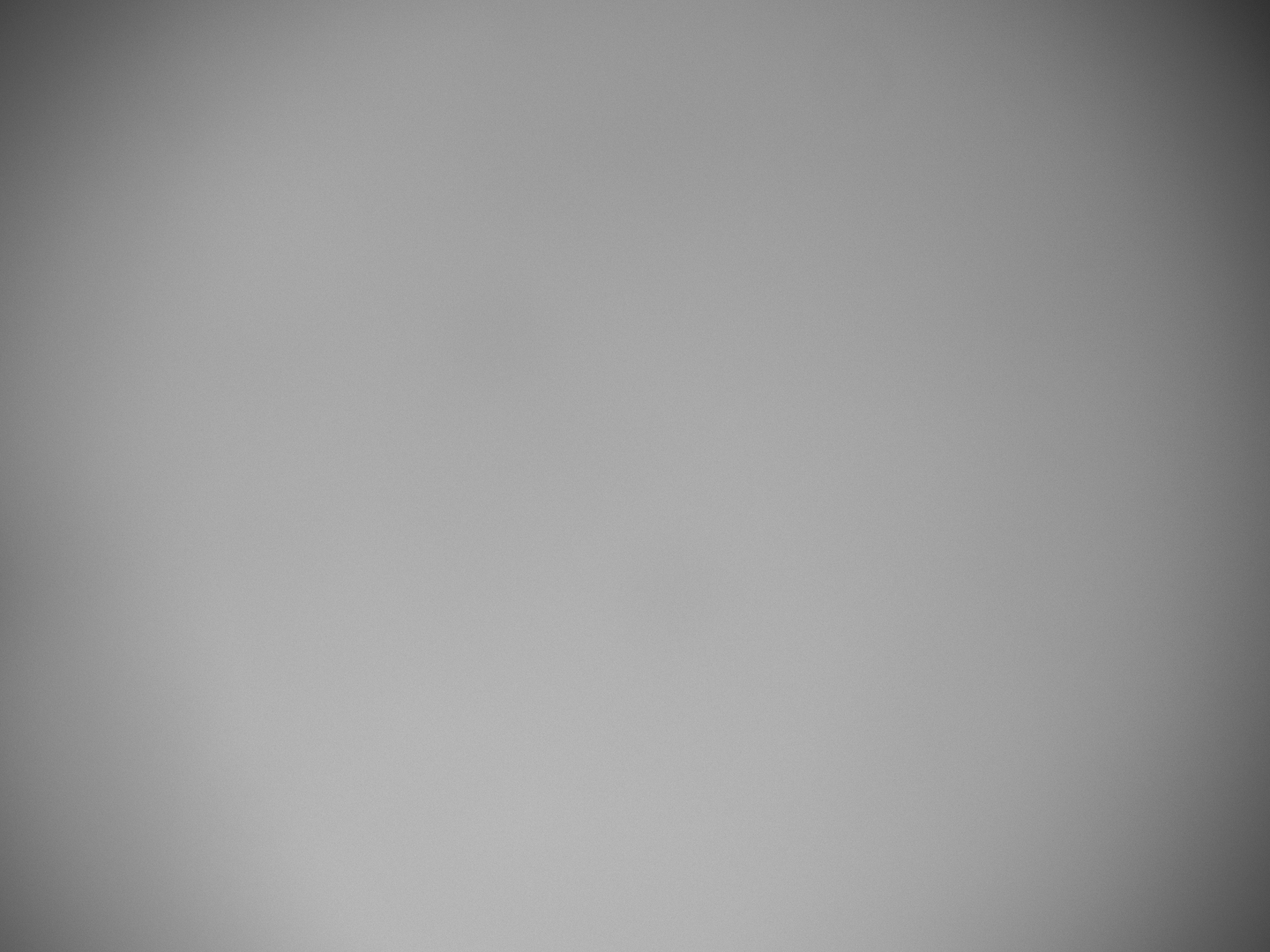** | **** | **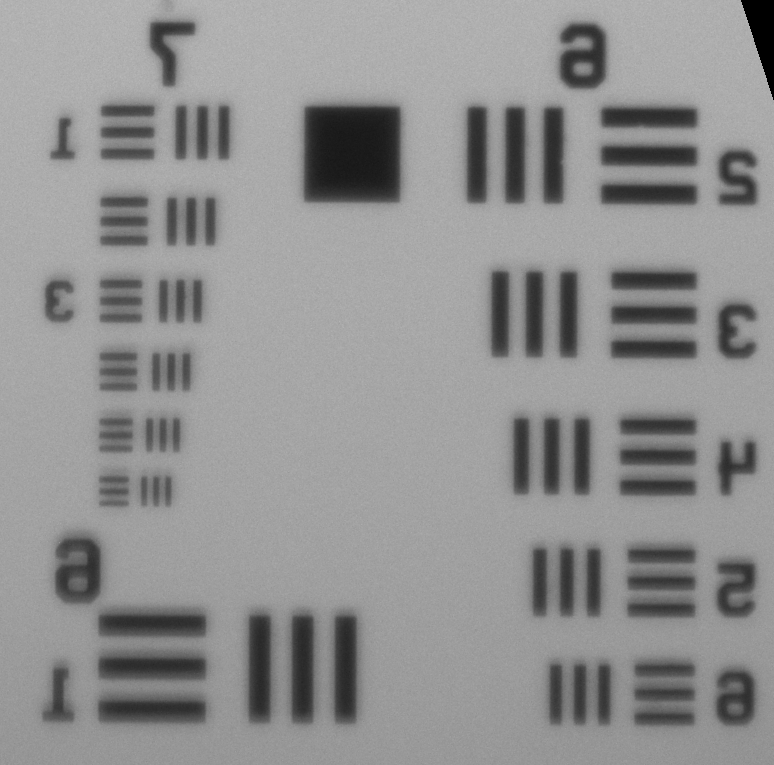** | **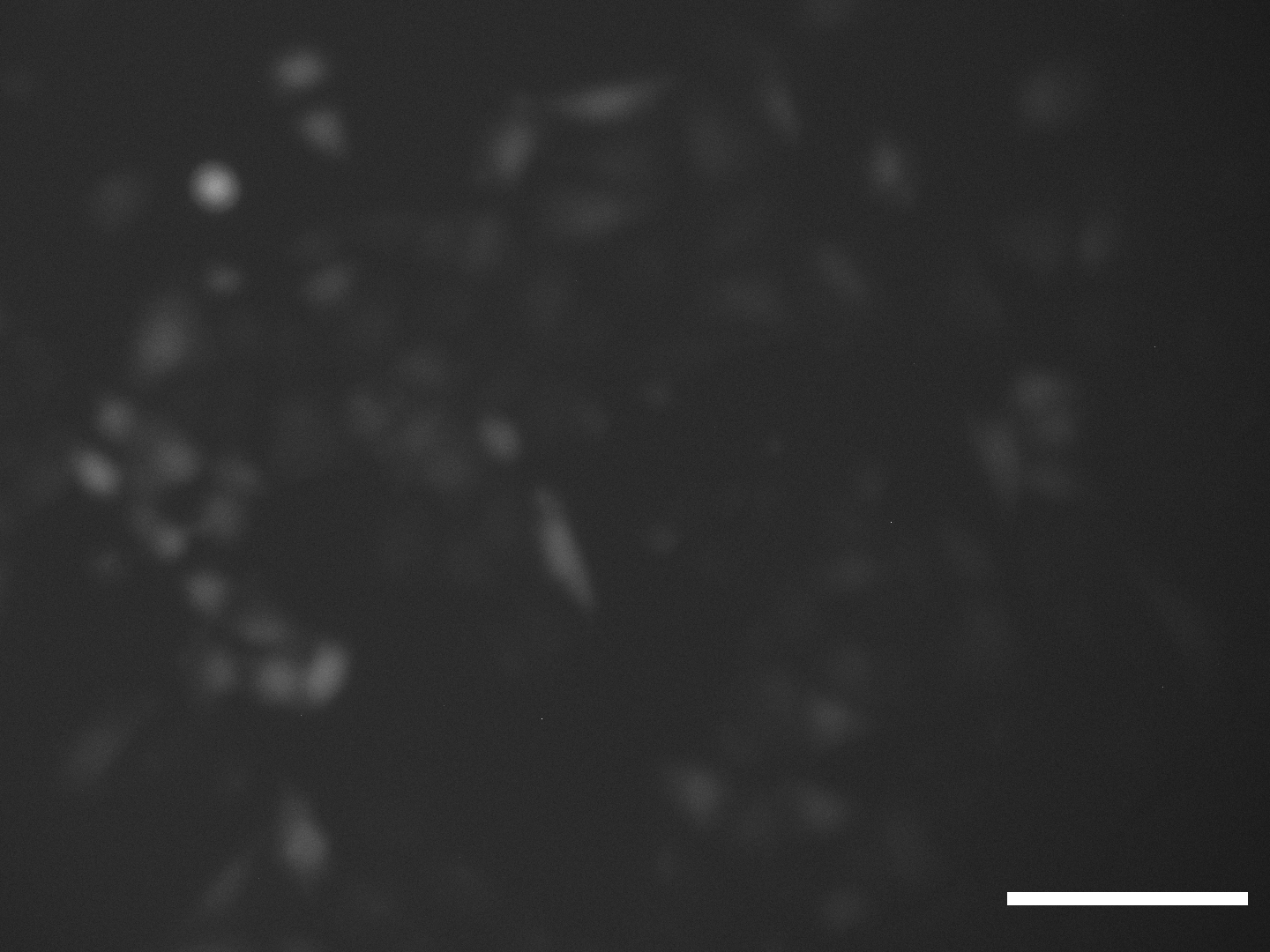** |
| Zeiss  EC Plan NEOFLUAR  20x NA0.5 | £  1400 | Yes | **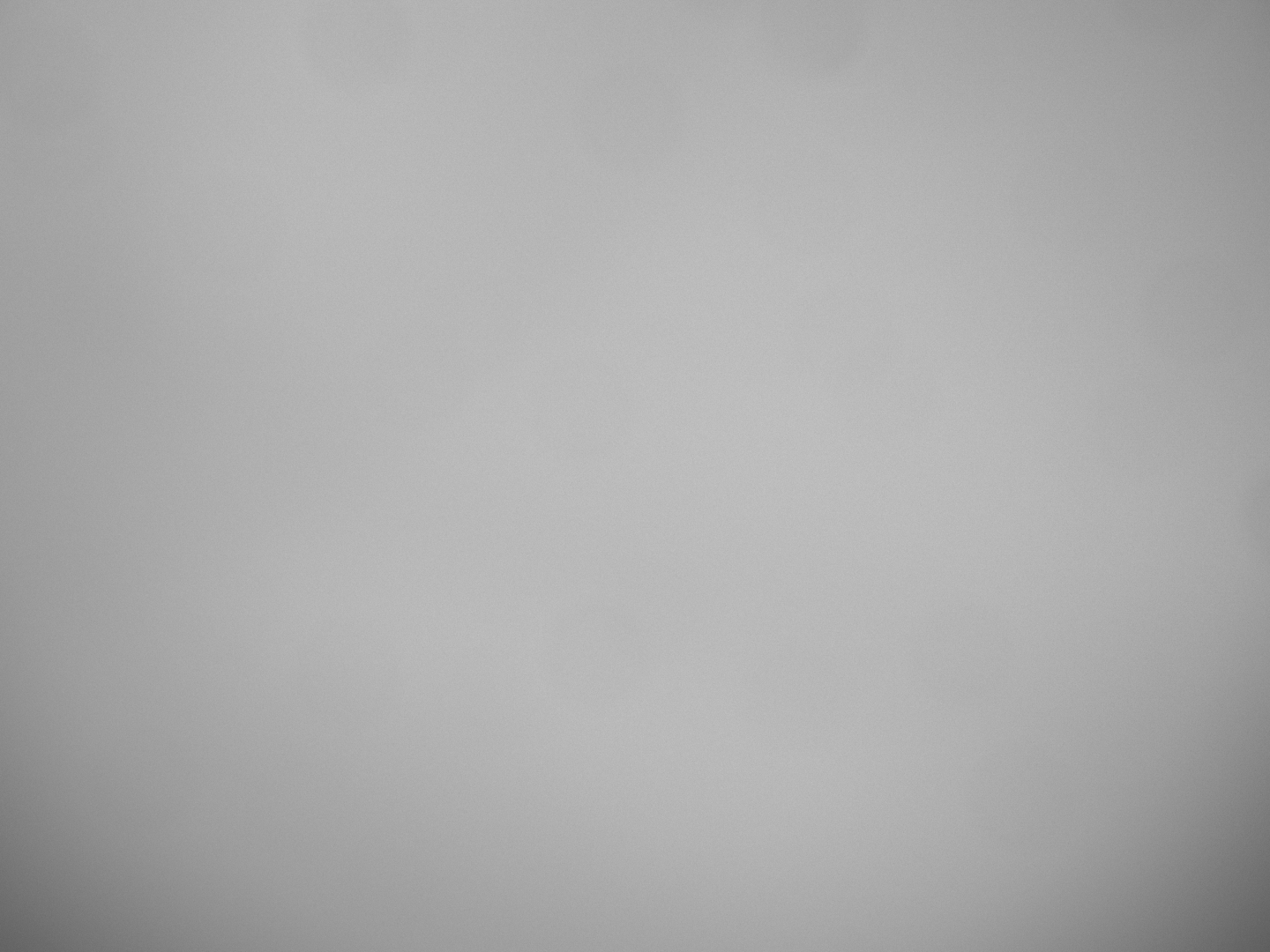** | **** | **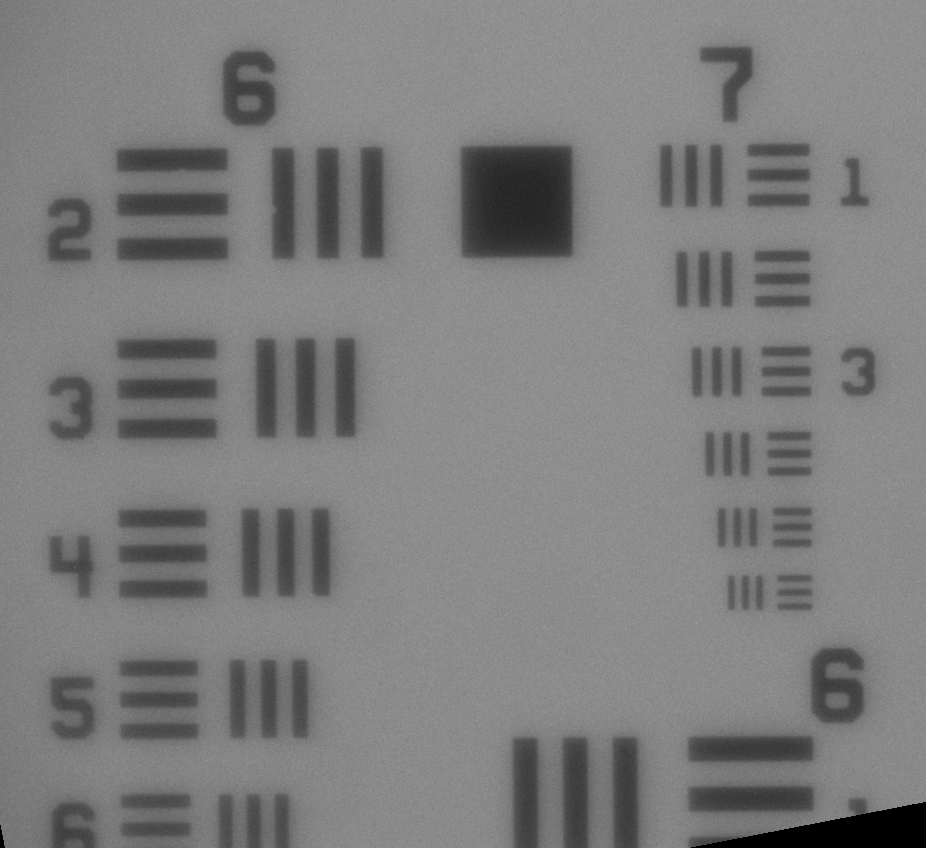** | **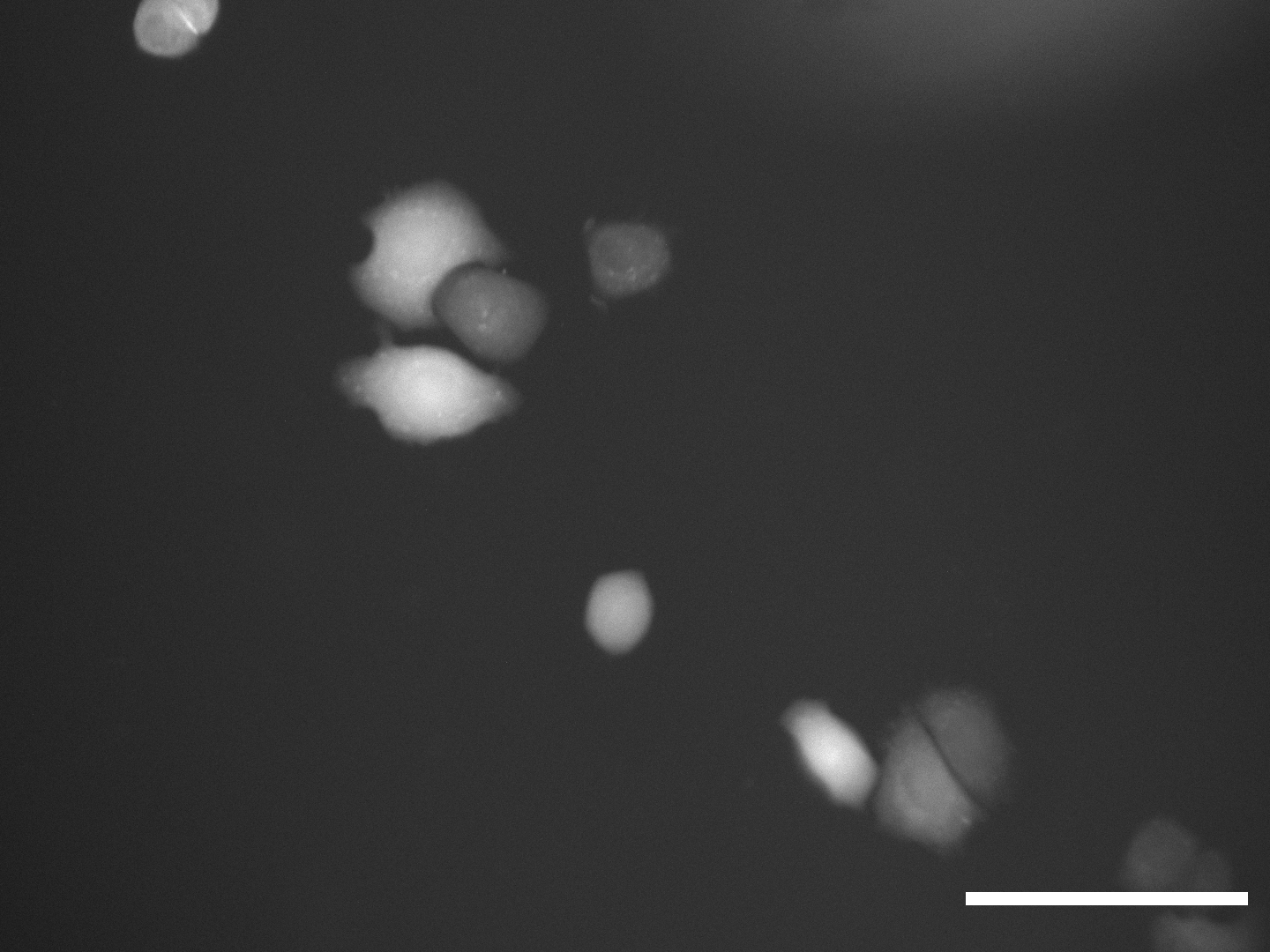** |
| Olympus LCPlanFl 20x | Used: £350-400 | Yes | **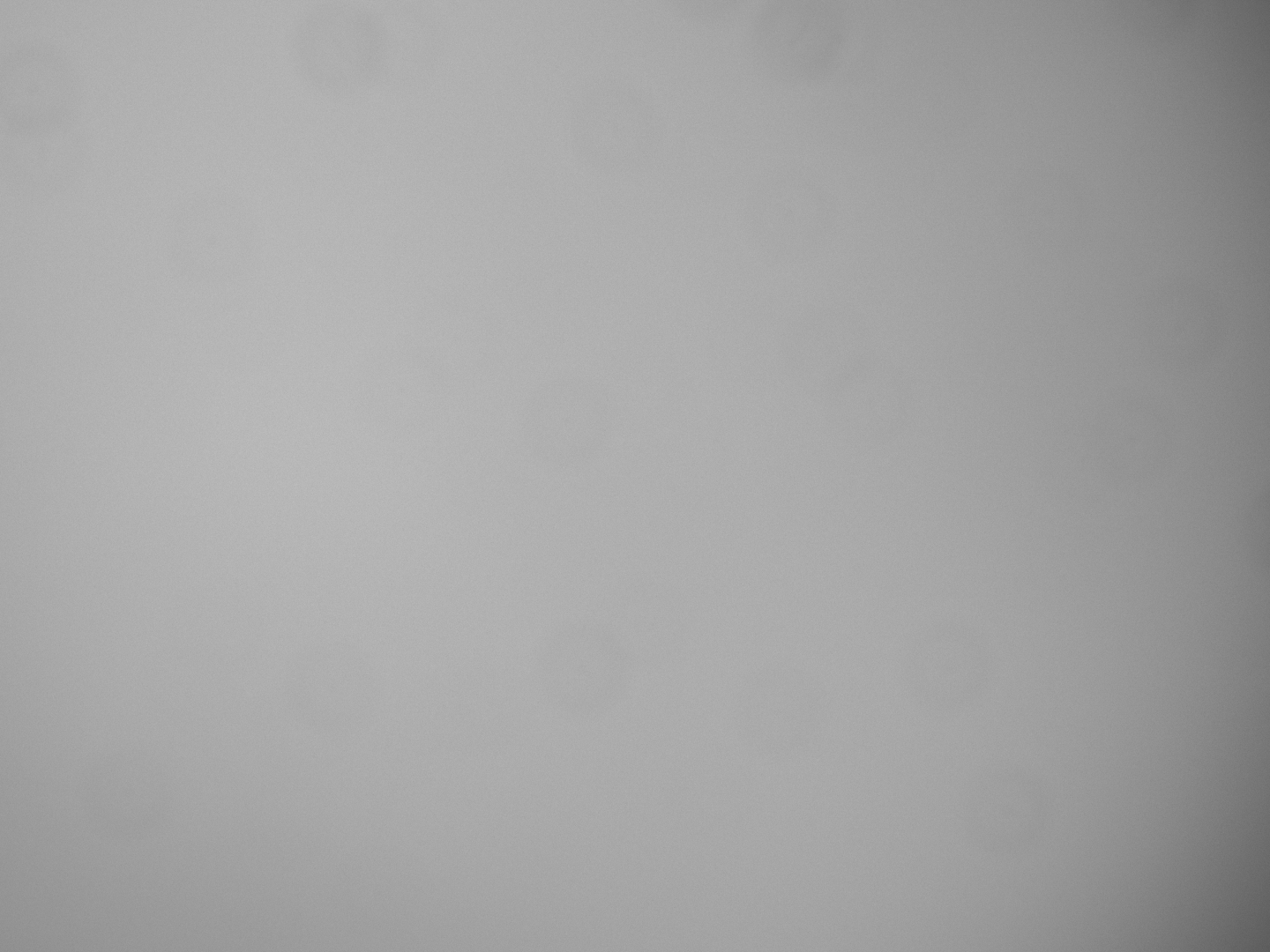** | **** | **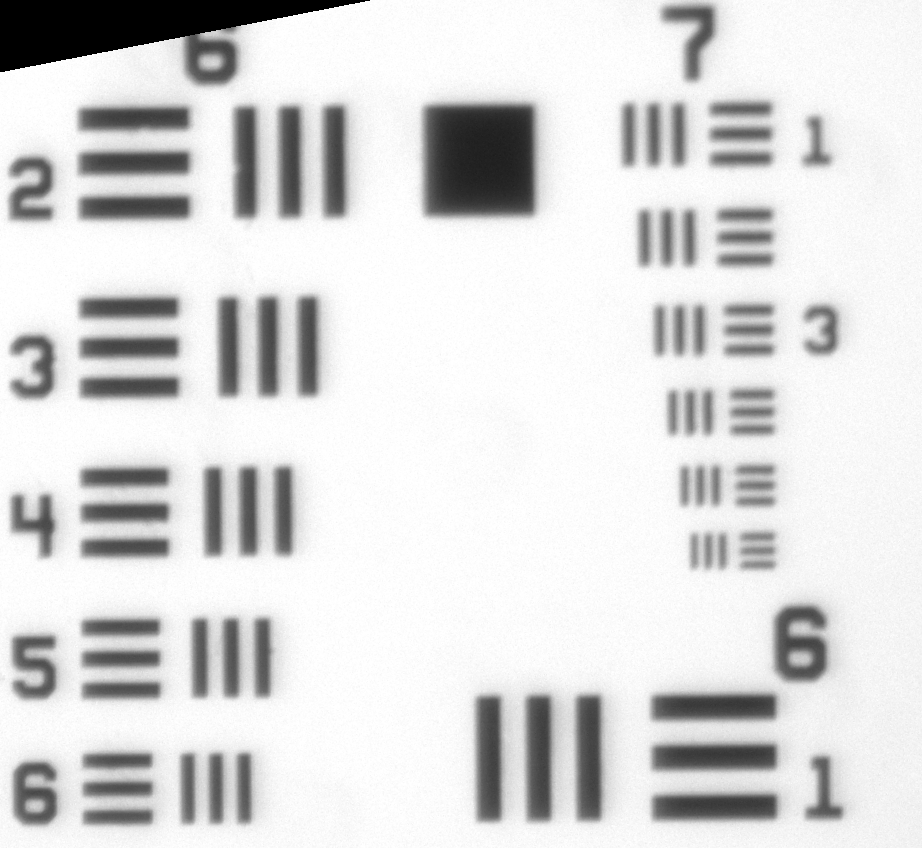** | **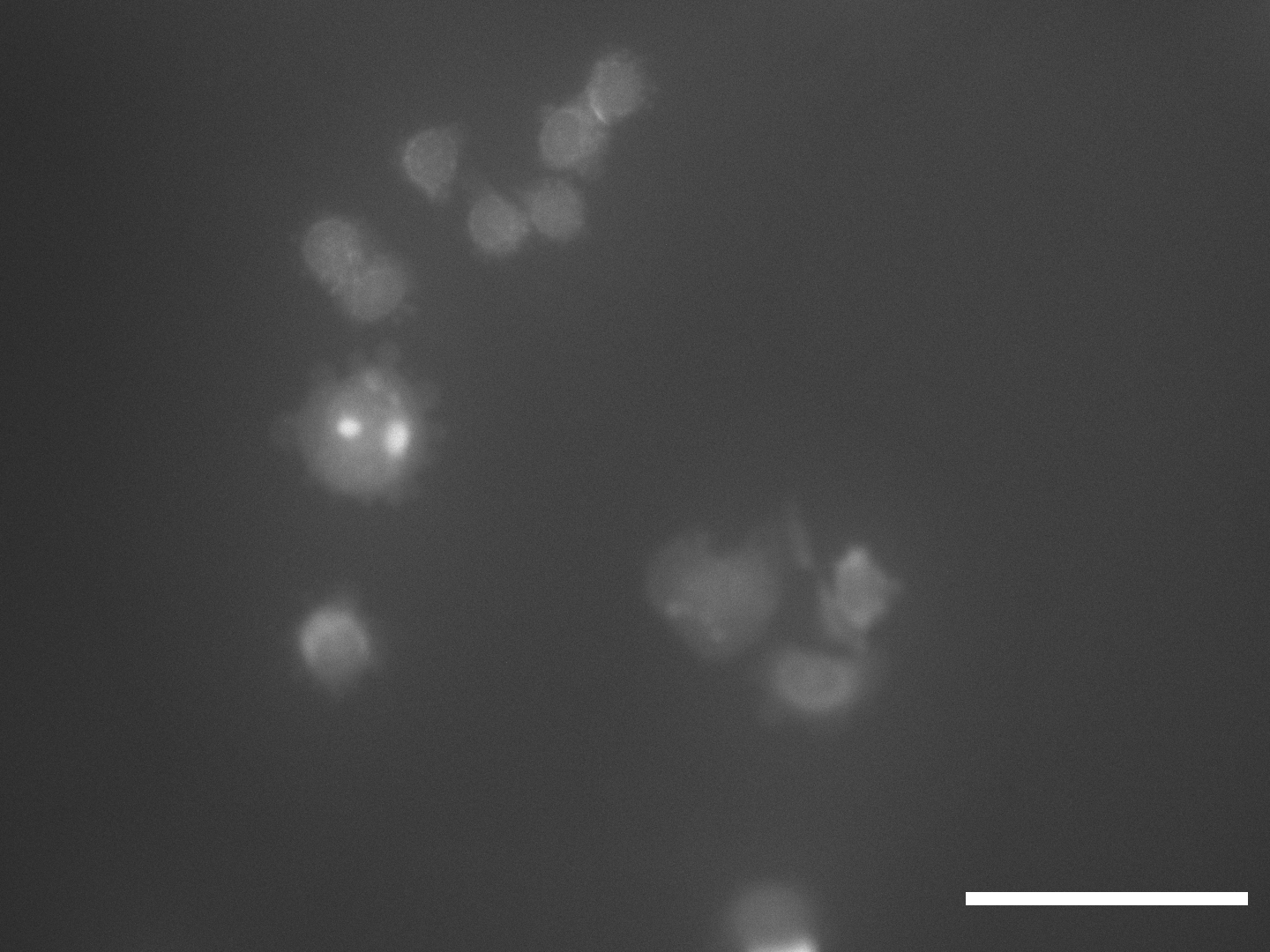** |
| Leica  C Plan L  20x/0.30 PH1 | Used:  £350-400 | Yes | **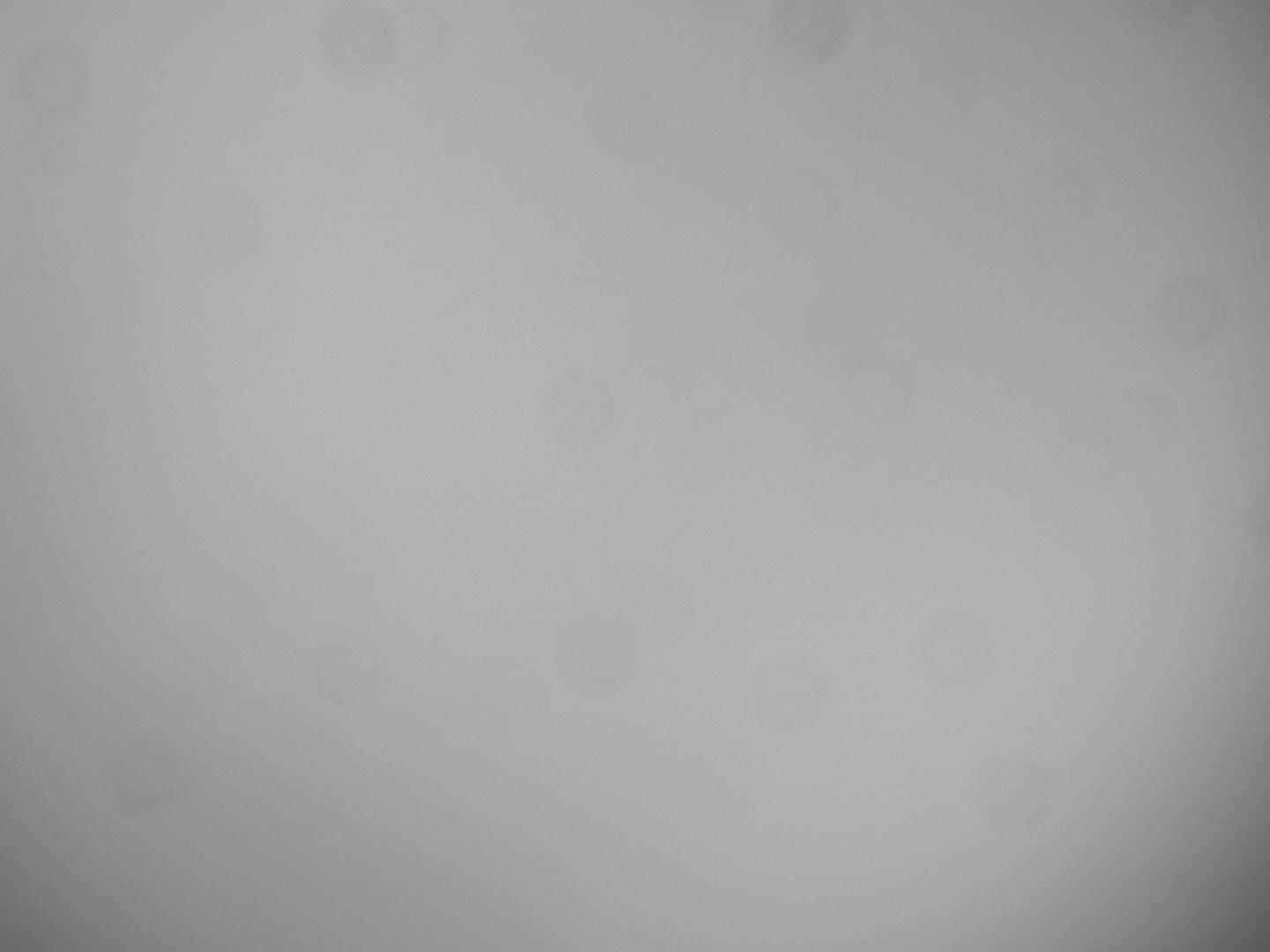** | **** | **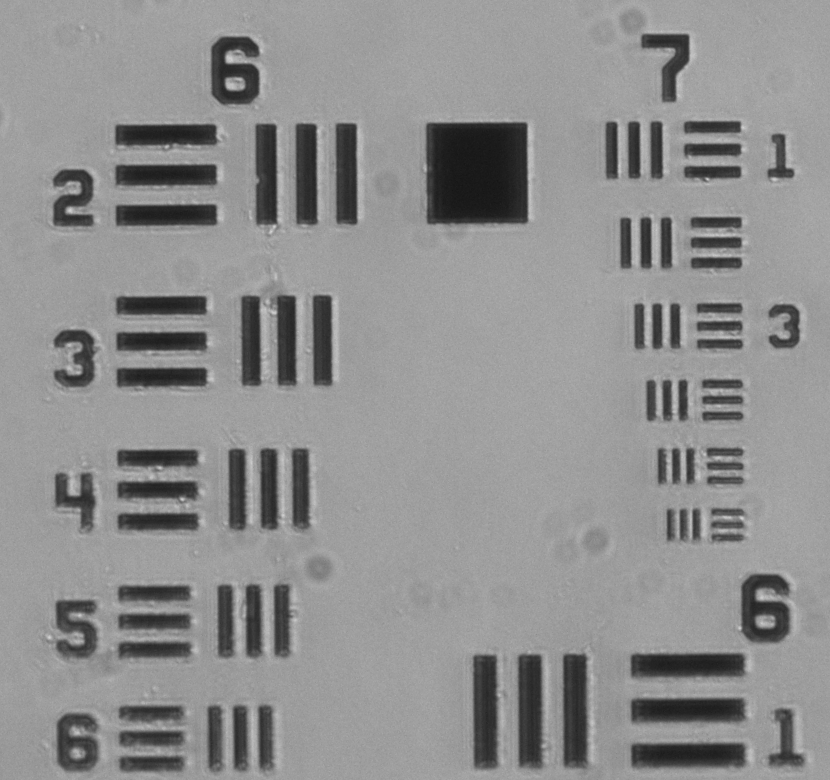** | **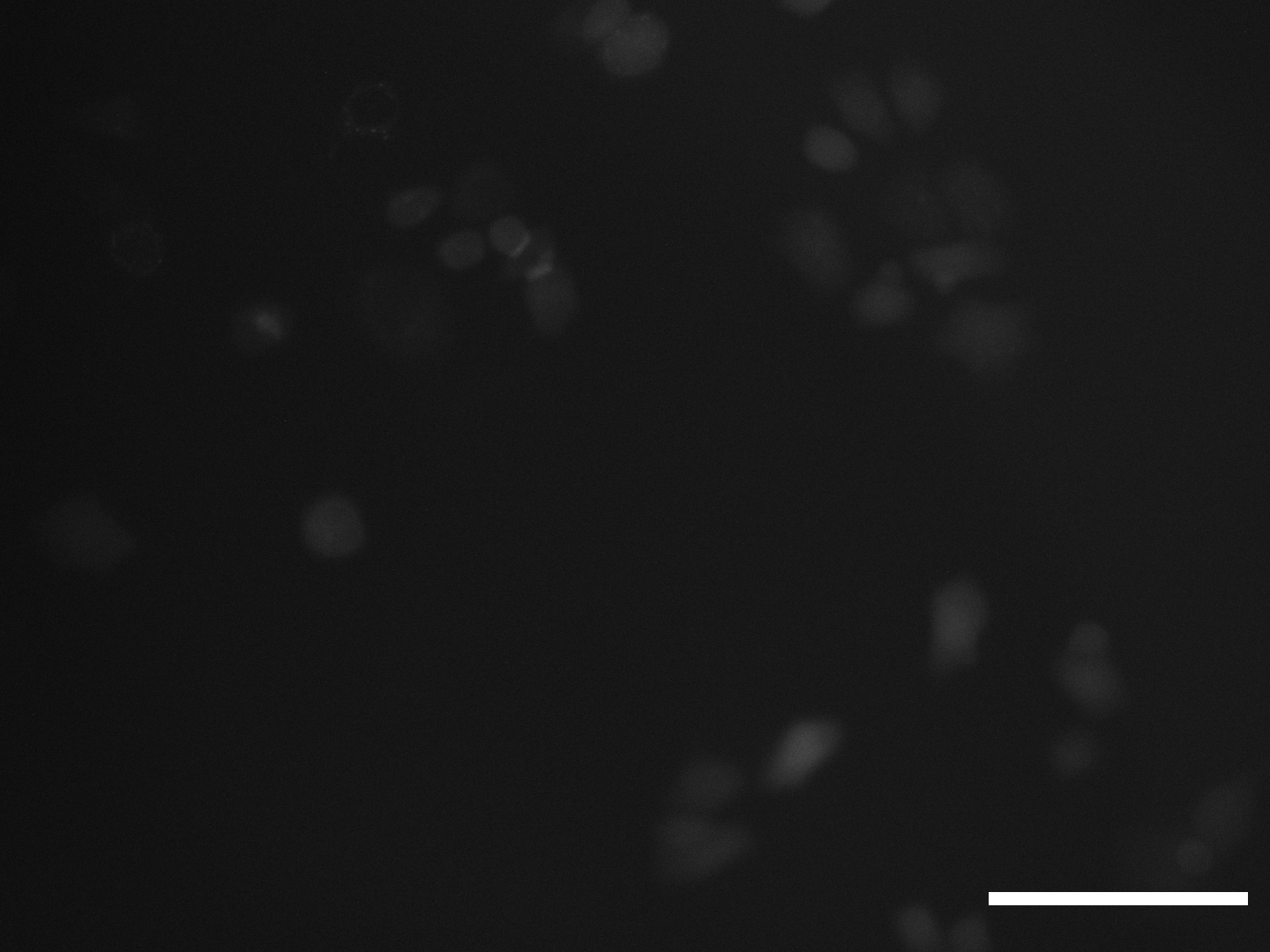** |

**Supplementary Table S2. Properties of different objectives tested with the developed imaging system.**

**SUPPLEMENTARY FIGURES**


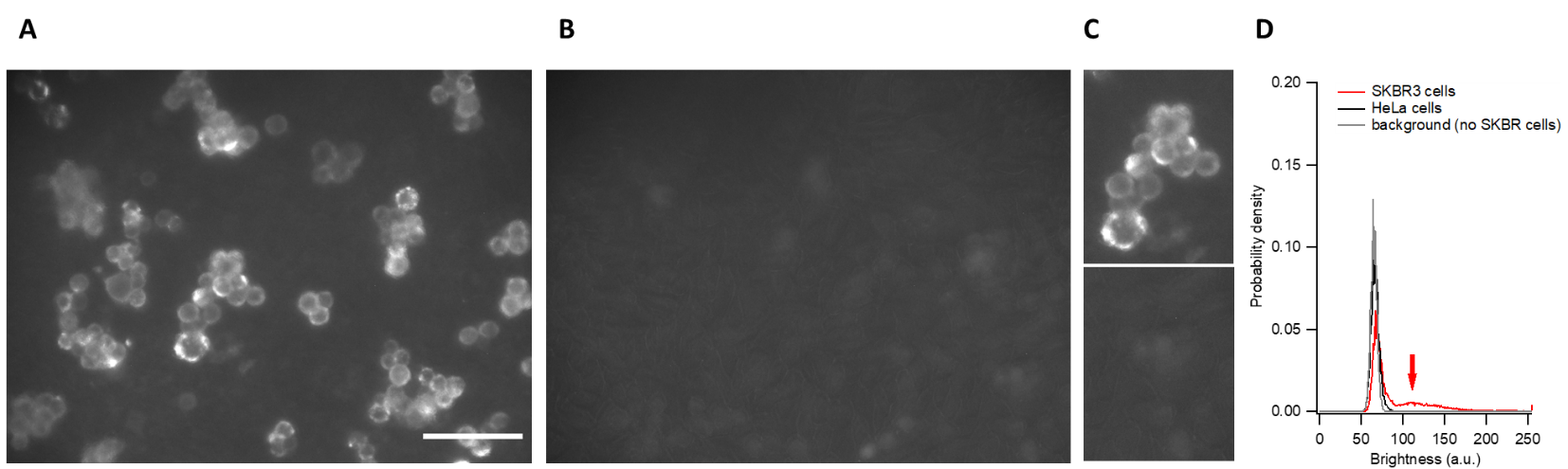


**Supplementary Fig. S1. Calculation of fluorescent distribution of SKBR and HeLa cells labelled with Herceptin.** **A, B-** SKBR cells and HeLa cells respectively. **C-** parts of images used for calculation of fluorescent distribution. **D-** Distribution of fluorescence of SKBR cells (red), HeLa cells (black) and background fluorescence (grey). Second peak (shown by arrow) represents immunofluorescence. Overall fluorescence was 91.9 ± 35.6 (Mean ± SD) for SKBR cells and 66 ± 4.9 for HeLa cells. Scale bar 100µm.


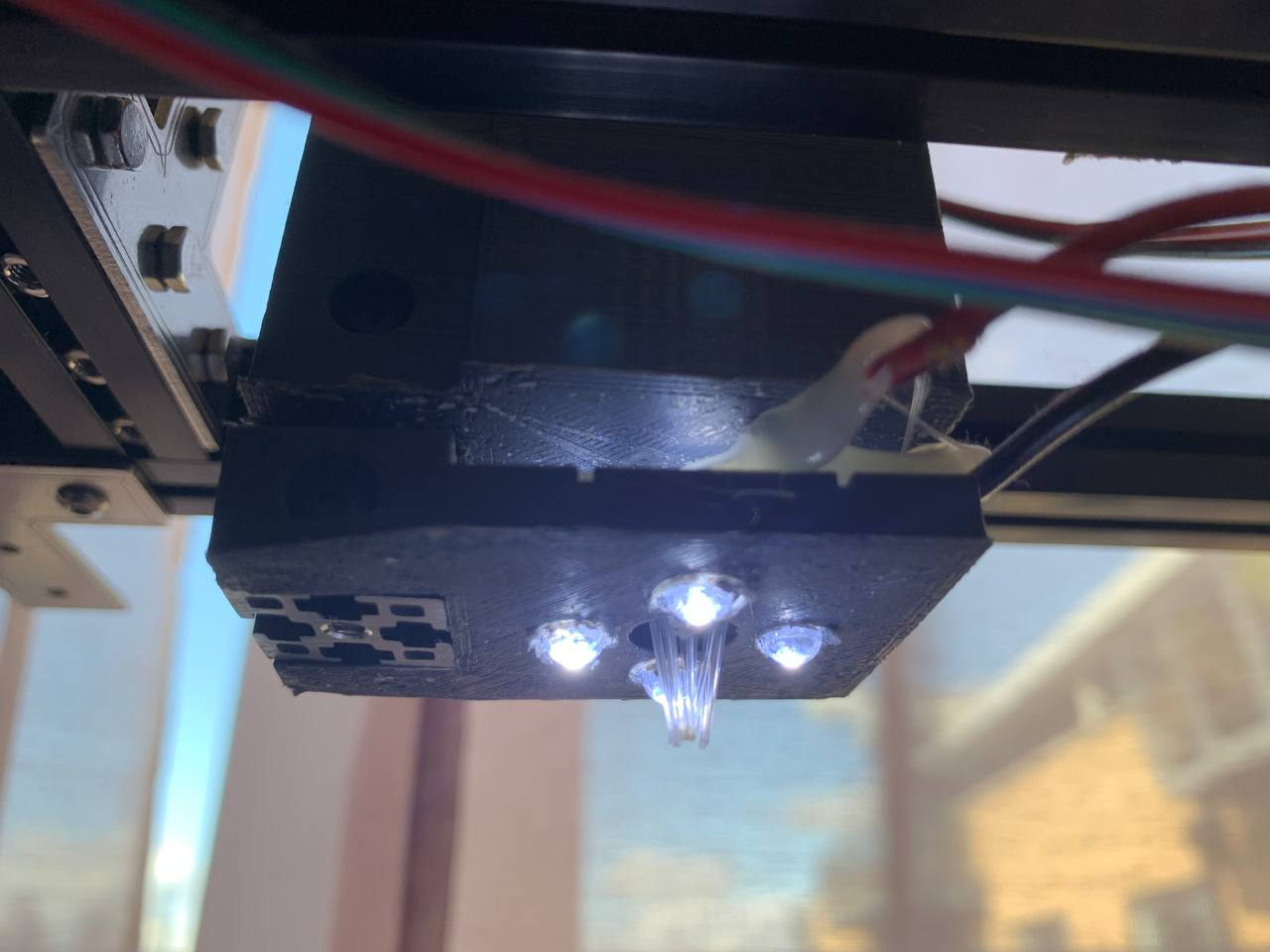


**Supplementary Figure S2. Solution application manifold, bottom view.**


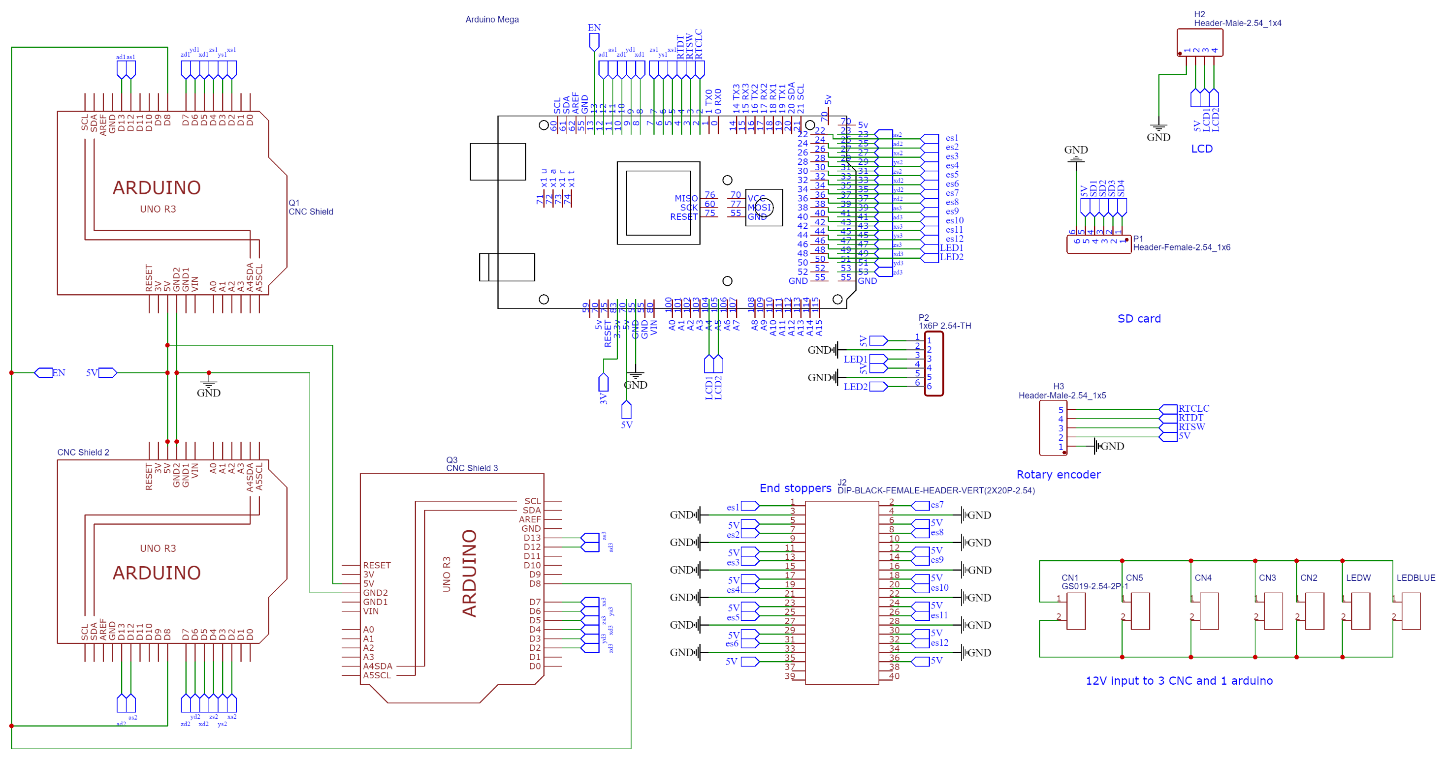


**Supplementary Figure S3. The electric circuit operating the main hardware.** The main circuit consists of slots for three CNC Shields, Arduino Mega, 12 inputs for end switches. 3 CNC shields (left) drive 12 Nema 17 motors. All 12 motor drivers are set to drive 1/8th step. All three CNC shields are connected to Arduino Mega (top) multiple pins. Bottom – 24 pins are used for 8 endstoppers (3 pins each). The full scheme also containing 12 Volt input from the power source to drive all three CNC shields and LEDs as well as pins reserved for driving LCD screen, SD card and a rotary encoder is shown in Supplementary Figure 3


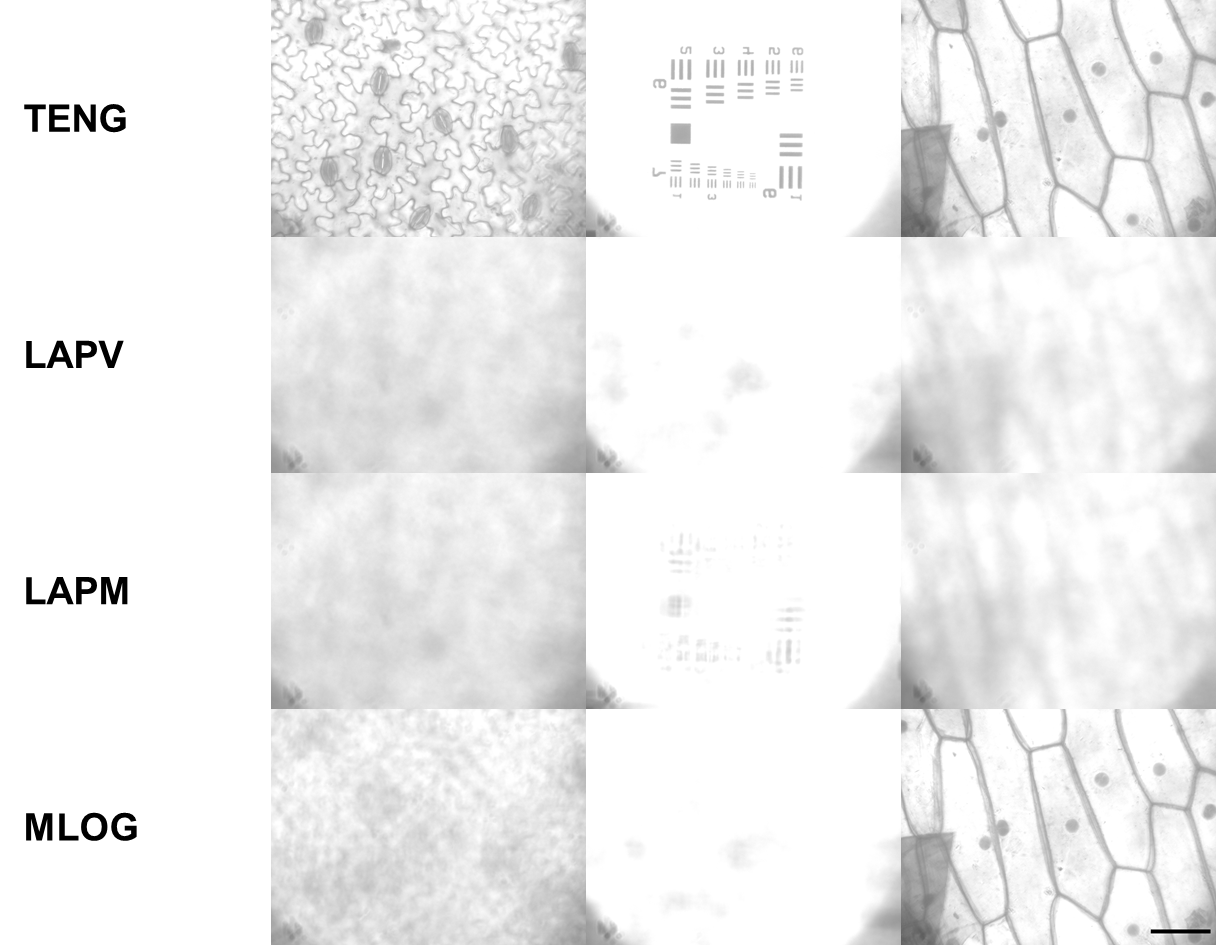


**Supplementary Figure S4. Examples of autofocusing with 4 different focus measures and 3 different microscopy slides.** Middle slide is USAF1951 standard. Scale bar - 100µm


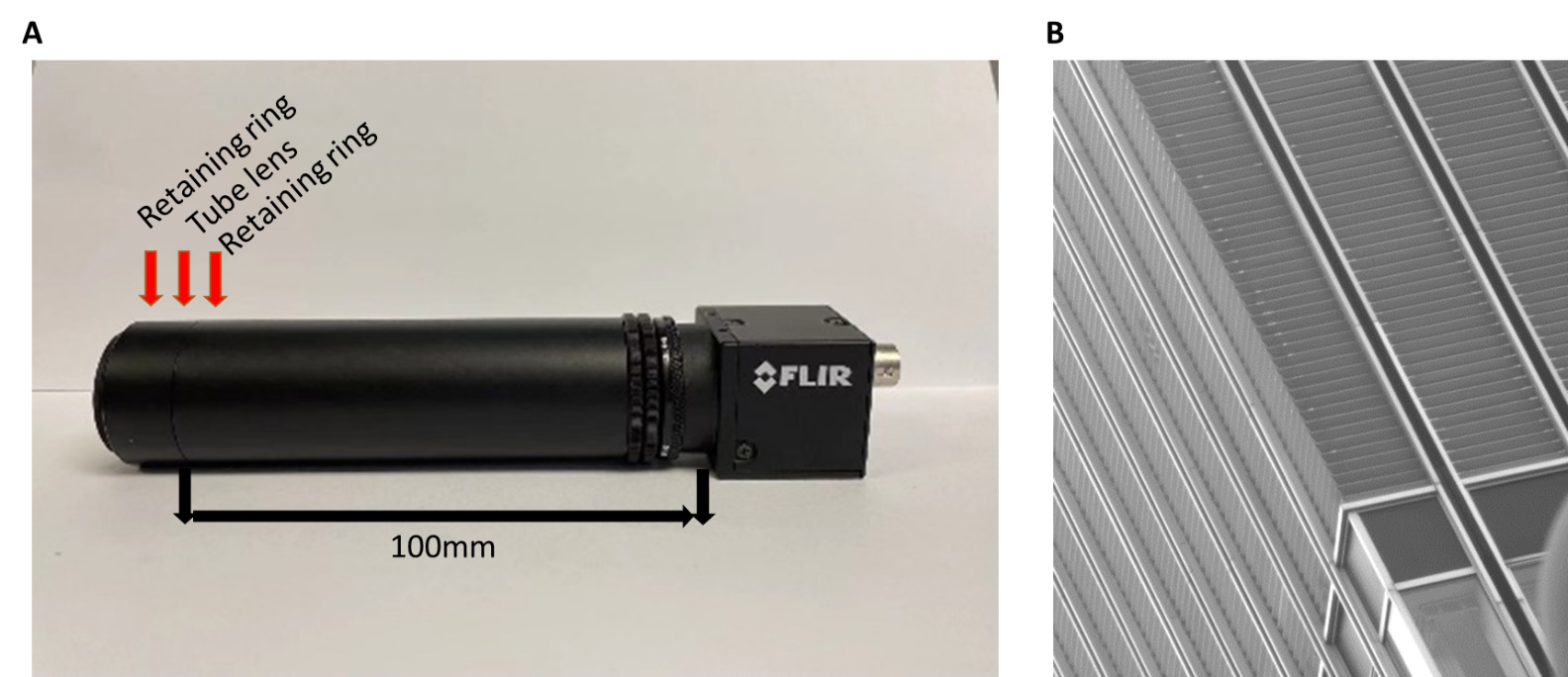


**Supplementary Figure S5. Positioning of the camera in the focus of the 100mm tube lens. A-** part of the microscope with the tube lens and the camera. The tube lens is positioned between two retaining rings ~10mm away from the end of the tube and is loated precisely 100mm from the camera sensor. Camera is connected to SM1 tube using SM1T2 coupler allowing to vary the distance between the camera and the lens. **B-** example of an image when distant object (located ~200m away) is in focus.


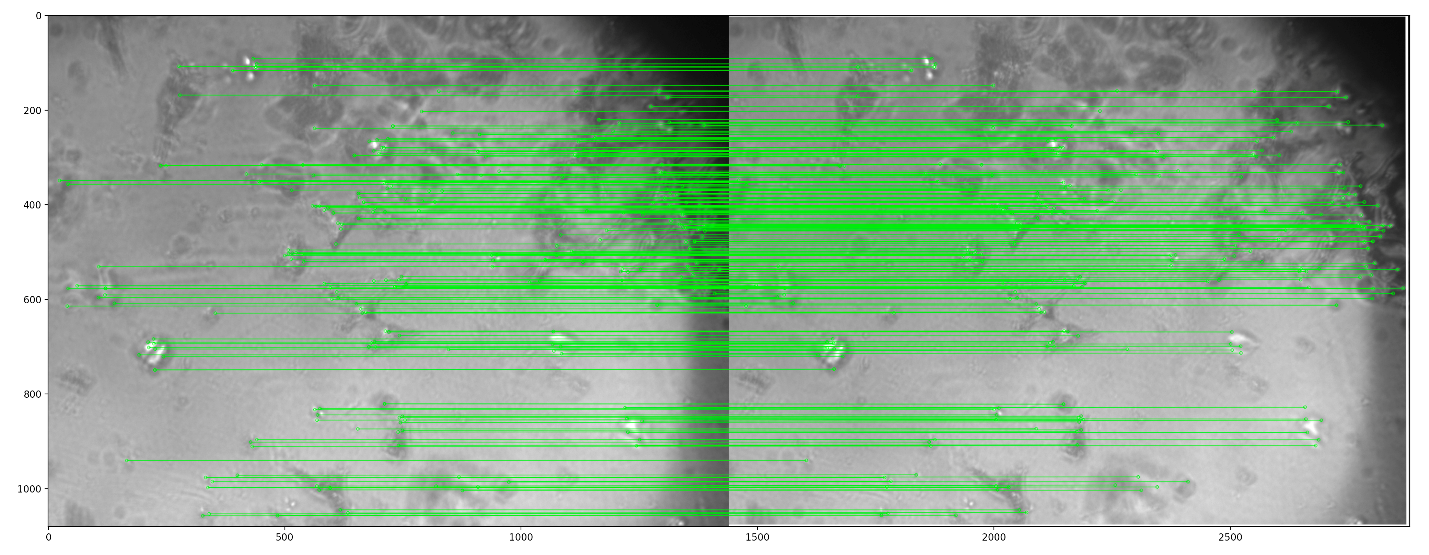


**Supplementary Figure S6. Calculation system vibration using SIFT and RANSAC algorithms.** Two frames were subjected to scale invariant feature transform (SIFT) that detects the key points of each image (green circles). Then the RANSAC algorithm was used to compare the position of each point.

**Supplementary Videos**

**Supplementary Video 1. Example of autofocusing performed in visible light.**

<https://www.youtube.com/watch?v=Sm55gbzcKX8>

**Supplementary Video 2. Example showing the work of the perfusion system.**

<https://www.youtube.com/watch?v=OPwA0h9CPLs>

**Supplementary Video 3. Example of calcium imaging performed on multiple wells**. See Fig.1 for more information. Note that these experiments were performed sequentially.

<https://www.youtube.com/watch?v=2gE3afugPMA>

**Supplementary Video 4.** **Example of vibrations in the system when the perfusion manifold is moving up and down.**

[**https://www.youtube.com/watch?v=iIMHp3cgBOw**](https://www.youtube.com/watch?v=iIMHp3cgBOw)
